# Development of a potent benzonitrile-based inhibitor of glutaminyl-peptide cyclotransferase-like protein (QPCTL) with antitumor efficacy

**DOI:** 10.1038/s41392-023-01715-x

**Published:** 2023-12-15

**Authors:** Lei Yu, Pengcheng Zhao, Yaoliang Sun, Zening Zheng, Wenhao Du, Lishan Zhang, Yaxu Li, Longyan Xie, Shilin Xu, Ping Wang

**Affiliations:** 1 Tongji University Cancer Center, Shanghai Tenth People’s Hospital, School of Medicine, Tongji University, Shanghai, China; 2 Department of Medicinal Chemistry, Shanghai Institute of Materia Medica, Chinese Academy of Sciences, Shanghai, China

**Keywords:** Target validation, Drug development

**Dear Editor**,

Immune checkpoint therapies manipulating the immune system to eliminate tumor cells have shown remarkable clinical efficacy in treating various cancers. CD47, an emerging efficient immune checkpoint, is crucial for cancer cells to evade macrophage-mediated phagocytosis by interaction with signal-regulatory protein α (SIRPα). Antibodies blocking the CD47/SIRPα interaction have been effective to promote macrophage-mediated phagocytosis in various types of cancer in mice and humans. CD47 is not only highly expressed in tumor cells, but also normal cells, such as red blood cells (RBCs). Thus, during clinical trials involving cancer patients, anti-CD47 antibodies may promote the macrophages-mediated phagocytosis of RBCs, ultimately inducing undesirable anemia side effects. In contrast, small molecule inhibitors interrupting CD47/SIRPα axis have shown potential to overcome the anemia, possibly due to their lower immunogenicity and shorter half-life compared to antibodies.^1^ Hence, developing the novel strategies, especially those without the anemia side effect, to intervene in CD47/SIRPα interaction will benefit cancer immunotherapy.

Recent studies from both Schumacher^2^ and our group^3^ reported that the formation of pyroglutamate on CD47 mediated by glutaminyl-peptide cyclotransferase like protein (QPCTL or isoQC) is essential for its binding to SIRPα and function as “don’t eat me” signal.^2,3^ QPCTL deficiency significantly enhances the macrophage-mediated phagocytosis of tumor cells.^2,3^ Moreover, inhibition of QPCTL can enhance the efficacy of PD-1 blockade via reshaping the infiltration of myeloid cells.^4^ These studies indicate that QPCTL is an attractive target for the treatment of cancers.^4^

QPCTL, an isoenzyme of glutaminyl-peptide cyclotransferase (QPCT), catalyzes the cyclization of N-terminal glutamine and glutamic acid residues on target proteins such as CCL2, CCL7 and CX3CL1, forming pyroglutamate residues. In recent decades, small molecule inhibitors targeting QPCT have been developed for Alzheimer’s disease treatment, and some of these inhibitors, including PBD150 (**1**), PQ912 (**2**) and SEN177 (**3**) (supplementary Fig. S1), also exhibited inhibitory activity against QPCTL. However, research on QPCTL inhibitors for tumor immunotherapy is still in its early stages, highlighting the need for the novel and potent QPCTL inhibitors. To this end, we designed a series of QPCTL inhibitors utilizing a structure-based approach starting from SEN177 (supplementary Table S1 and S2). Although the co-crystal structure of SEN177 bound to QPCTL is unavailable, we postulated that SEN177 binds to QPCTL similarly to QPCT due to the highly conserved structure shared between their active sites. In the binding model of SEN177 and QPCT, a notable feature is the nitrogen atom in the pyridine core that forms a hydrogen bond interaction with the backbone NH of Gln304 mediated by a structural water molecule (supplementary Fig. S2a). To improve binding affinity, we replaced the nitrogen atom in the pyridine core of SEN177 with a nitrile group to create benzonitrile-containing compound **4** (**QP5020**) (Fig. 1a and supplementary Fig. S2b), since mimicking or displacing a binding-site water molecule is a well-established strategy. Our computational binding model showed that the nitrile group in **QP5020** forms a hydrogen bond with the Glu325 residue (supplementary Fig. S2c). Subsequently, we assessed the inhibitory activity of **QP5020** against QPCTL and our results demonstrated that **QP5020** was 8.7-fold more potent than SEN177, with an IC_50_ value of 15.0 ± 5.5 nM against QPCTL (Fig. 1b). Further optimization efforts led to a highly potent compound **28** (**QP5038**), exhibiting an IC_50_ value of 3.8 ± 0.7 nM against QPCTL (Fig. 1b) and a comparable inhibition to QPCT (supplementary Fig. S3), suggesting that **QP5038** have the potential for more disease treatments other than cancer. The remarkable potency warrants further characterization of **QP5020** and **QP5038** as QPCTL inhibitors.

**Fig. 1:**
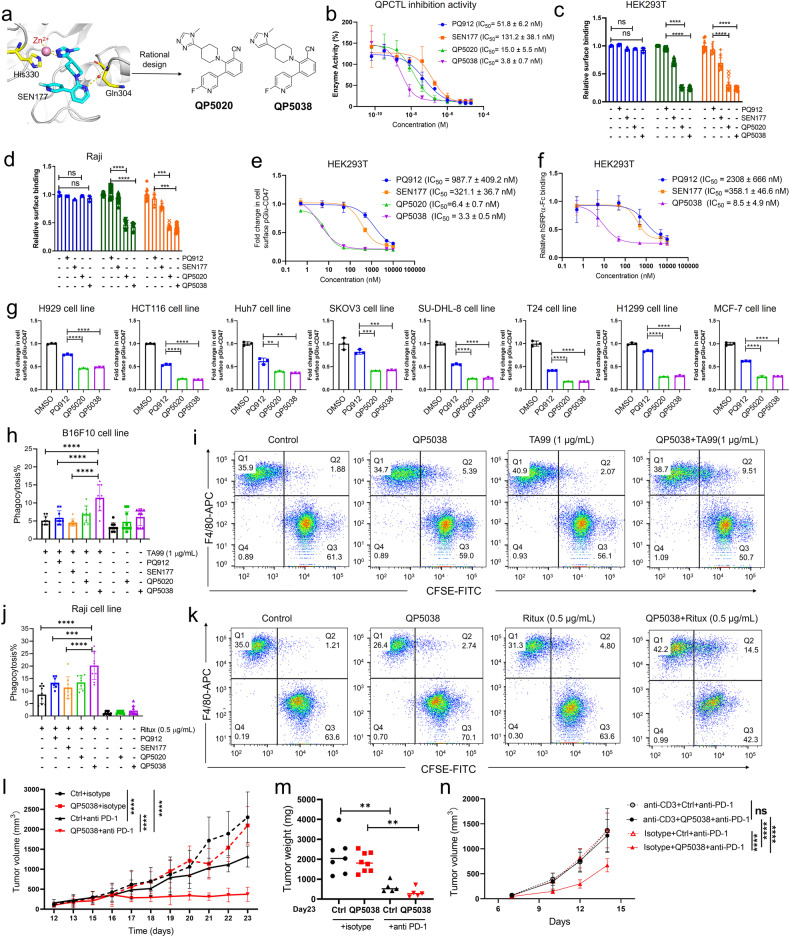
Discovery of **QP5038** as potent QPCTL inhibitor. **a** Design of novel and potent QPCTL inhibitors. QPCT protein structure (PDB: 6GBX) is downloaded from PDB protein structure database. **b** Fluorescent assay determination of IC_50_ values of inhibitors against QPCTL. Data represent *n* = 3 biological replicates and mean ± SD. **c** Cell surface binding of anti-human CD47 antibody clone hCD47-B6H12, hCD47-CC2C6 and human hSIRPα-Fc to HEK293T cells after treatment with 100 nM QPCTL inhibitors for 48 h, as determined by flow cytometry. **d** Cell surface binding of anti-human CD47 antibody clone hCD47-B6H12, hCD47-CC2C6 and human hSIRPα-Fc to Raji cells after treatment with 100 nM QPCTL inhibitors for 48 h as determined by flow cytometry. In **c** and **d**, values indicated mean fluorescence intensity (MFI) relative to cells stained with DMSO. Data represent *n* = 3 biological replicates and mean ± SD of triplicates. Statistically significant differences were determined by one-way ANOVA, ∗∗∗*p* < 0.001, ∗∗∗∗*p* < 0.0001. **e** Dose-dependent inhibition of pGlu-CD47 following treatment with QPCTL inhibitors for 48 h in HEK293T cells. **f** Dose-dependent inhibition of cell surface binding of human hSIRPα-Fc to HEK293T cells following treatment with QPCTL inhibitors for 48 h. In **e** and **f**, data represent *n* = 3 biological replicates and mean ± SD of triplicates. **g** Cell surfac**e** binding of anti-human CD47 antibody clone hCD47-CC2C6 to different cells, such as myeloma (H929), colon cancer (HCT116), hepatocellular carcinoma (Huh7), ovarian adenocarcinoma (SKOV3), lymphoma (SU-DHL-8), bladder cancer (T24), lung cancer (H1299), and breast cancer (MCF-7), after treatment with 500 nM QPCTL inhibitors for 48 h, as determined by flow cytometry. Data are representative of three independent experiments. Statistically significant differences were determined by unpaired two-tailed t-test, ∗∗*p* < 0.01, ∗∗∗*p* < 0.001, ∗∗∗∗*p* < 0.0001. **h**, **i** Phagocytosis of control-treated (DMSO) (-) or QPCTL **i**nhibitors-treated (+) B16F10 cells in the presence or absence of the anti-mouse TRP1 antibody TA99 by mouse macrophages following treatment with 10 μM inhibitors for 48 h. **j**, **k** Phagocytosis of control-treated (DMSO) (-) or QPCTL inhibitors-treated (+) Raji cells in the presence or absence of the anti-human CD20 antibody rituximab (Ritux) by mouse macrophages following treatment with 10 μM inhibitors for 48 h. Phagocytosis was determined by the number of the CFSE ^+^ labelled F4/80^+^ macrophages vs the total tumor cells, and data are mean values of three biological experiments in **h**, **i**, **j** and **k**. The presented data is a representative image from three independent experiments with similar results in **i** and **k**. Statistically significant differences were determined by one-way ANOVA, ∗∗∗*p* < 0.001, ∗∗∗∗*p* < 0.0001 in **h**, **i**, **j** and **k**. **l** Anti-tumor efficacy of **QP5038** with once daily dosing at 25 mg/kg in the presence or absence of the anti-PD-1 antibody. The total study length was 24 days. Statistically significant differences were determined by two-way ANOVA, ∗∗∗∗*p* < 0.0001. **m** Quantification of xenografted tumor weight when mice are sacrificed. The data were presented as the mean ± SD and statistically significant differences were determined by two-way ANOVA, ∗∗*p* < 0.005. **n** Anti-tumor efficacy of **QP5038** in the presence or absence of the T cell depletion antibody. Statistically significant differences were determined by two-way ANOVA, ∗∗∗∗*p* < 0.0001, ns not significant

To investigate the effects of our QPCTL inhibitors on CD47 pyroglutamation (pGlu-CD47), we treated HEK293T and lymphoma Raji cells with **QP5020** and **QP5038**. Our data demonstrated that both compounds showed superior inhibitory effects on CD47 pyroglutamation compared to SEN177 and PQ912 at a concentration of 100 nM, whereas did not alter the overall cell surface levels of CD47 (Fig. 1c, d). Moreover, **QP5020** and **QP5038** exhibited dose-dependent inhibition of pGlu-CD47 levels in HEK293T cells with remarkable IC_50_ values of 6.4 ± 0.7 nM and 3.3 ± 0.5 nM, respectively (Fig. 1e). Importantly, we found that both **QP5020** and **QP5038** significantly reduced the binding of human or mouse SIRPα protein to cell surface in HEK293T and tumor cells (Fig. 1c, d and supplementary Fig. S4), indicating that **QP5020** and **QP5038** can block the binding of CD47/SIRPα. Notably, **QP5038** attenuated the interaction of CD47/SIRPα in a dose-dependent manner with an IC_50_ value of 8.5 ± 4.9 nM in HEK293T cells, which is more potent than PQ912 and SEN177 (Fig. 1f). Our results also showed that **QP5038** did not markedly impair the cell viability (supplementary Fig. S5). Furthermore, **QP5020** and **QP5038** significantly attenuated CD47 pyroglutamation in various types of cancer cells (Fig. 1g).

Blocking the interaction of CD47/SIRPα is known to remarkably enhance the capacity of macrophages to eliminate tumor cells, especially in combination with other anti-cancer antibodies, such as anti-TRP1 antibody TA99 or anti-CD20 antibody rituximab. In order to evaluate the ability of our QPCTL inhibitors to promote the macrophage-mediated phagocytosis of cancer cells, we performed an in vitro phagocytosis assay. Our results showed that **QP5038** significantly boosted the phagocytosis of B16F10 cells in combination with TA99 treatment (Fig. 1h, i and supplementary Fig. S6a), or Raji cells synergized with rituximab (Fig. 1j, k and supplementary Fig. S6b). Our data showed that the efficiency of **QP5038** on phagocytosis was much better than that of SEN177 and PQ912 (Fig. 1h–k and supplementary Fig. S6). Recently, Schloesser et al reported that CD47-QPCTL axis was upregulated in senescent cells and thereby suppressed the macrophage-mediated apoptotic cells removal.^5^ We found that pretreatment with **QP5038** could block the inhibitory effect of senescent cells on macrophage-mediated phagocytosis of apoptotic cells (supplementary Fig. S7). In addition, **QP5038** also did not attenuate the cell viability and phagocytotic ability of macrophages (supplementary Fig. S8). These results further highlighted the potential of **QP5038** to enhance macrophage-mediated cancer cell clearance.

It was recently reported that QPCTL deficiency in tumor cells leads to an altered tumor microenvironment, which enhances the susceptibility of tumors to PD-1/PD-L1 blocking treatment. In light of this, we assessed the potential therapeutic effects of **QP5038** in combination with PD-1 inhibition in mice. Our data showed that the combination treatment of **QP5038** with anti-PD-1 antibody dramatically suppressed both tumor growth and tumor weight comparing to each single treatment and SEN177 treatment group without effect on mice body weight (Fig. 1l, m and supplementary Fig. S9). Furthermore, in vivo toxicity of **QP5038** was also assessed, revealing that **QP5038** would not cause an abnormal blood composition and organ damage in the indicated dosage (supplementary Fig. S10 and S11). Depletion of T cell in mice using anti-CD3 antibody blocked the enhancement of **QP5038** on anti-PD-1 antibody-mediated tumor inhibition (Fig. 1n and supplementary Fig. S12), suggesting that the anti-cancer effect of **QP5038** was due to the activation of immune response.

In short, we identified **QP5038** as a novel QPCTL inhibitor and has promising in vitro and in vivo anti-tumor efficacies. Our data support further investigation of **QP5038** as a potential clinical drug candidate for tumors, especially in combination with anti-PD-1 antibodies.

### Supplementary information

Supplementary materials

## Data Availability

All the data used for the current study are available from the corresponding author upon reasonable request.
