# Supplementary material for: Development of a potent benzonitrile-based inhibitor of glutaminyl-peptide cyclotransferase-like protein (QPCTL) with antitumor efficacy
Source: Signal Transduct Target Ther. 2023 Dec 15;8:454. doi: 10.1038/s41392-023-01715-x (PMC10721786; doi:10.1038/s41392-023-01715-x)
Supplement: Supplementary file 1 — Supplementary materials [file 41392_2023_1715_MOESM1_ESM.docx]

Supplementary materials for

**Development of a potent benzonitrile-based inhibitor of glutaminyl-peptide cyclotransferase-like protein (QPCTL) with antitumor efficacy**

Lei Yu^1#^, Pengcheng Zhao^1#^, Yaoliang Sun^2#^, Zening Zheng^1#^, Wenhao Du^2^, Lishan Zhang^2^, Yaxu Li ^1^, Longyan Xie^1^, Shilin Xu^2^*, Ping Wang^1^*

^#^These authors contributed equally: Lei Yu, Pengcheng Zhao, Yaoliang Sun, Zening Zheng

Correspondence:

Ping Wang, Email: wangp@tongji.edu.cn;

Shilin Xu, Email: slxu@simm.ac.cn

**This PDF file includes:**

**Supplementary tables 1 to 2**

**Supplementary figures S1 to S12**

**Materials and methods**

**1. Supplementary tables**

**Table 1.** *In vitro* activities for inhibition of QPCTL by compounds **4**-**25**.

| Compd. | R_1_ | QPCTL  IC_50_ (nM)^a^ |
| --- | --- | --- |
| PQ912 | -- | 51.8 ± 6.2 |
| SEN177 | -- | 131.2 ± 38.1 |
| **4** (QP5020) |  | 15.0 ± 5.5 |
| **5** |  | 56.5 ± 11.3 |
| **6** |  | > 1000 |
| **7** |  | > 1000 |
| **8** | H | > 1000 |
| **9** |  | 76.5 ± 21.3 |
| **10** |  | > 1000 |
| **11** |  | 417.1 ± 210.1 |
| **12** |  | 471.3 ± 243.7 |
| **13** |  | 368.0 ± 82.0 |
| **14** |  | 73.6 ± 11.5 |
| **15** |  | 123.9 ± 9.8 |
| **16** |  | > 1000 |
| **17** |  | 53.5 ± 11.8 |
| **18** |  | 74.5 ± 13.4 |
| **19** |  | 105.1 ± 22.3 |
| **20** |  | 186.5 ± 20.8 |
| **21** |  | 573.5 ± 85.5 |
| **22** |  | 17.6 ± 6.2 |
| **23** |  | 19.6 ± 12.5 |
| **24** |  | 135.3 ± 64.3 |
| **25** |  | > 1000 |

^a^Results are mean values of three independent experiments or mean ± SD unless specified otherwise.

**Table 2.** *In vitro* activities for inhibition of QPCTL by compounds **26**-**29**

| Compd. | R_2_ | QPCTL  IC_50_ (nM)^a^ |
| --- | --- | --- |
| **26** |  | 94.3 ± 13.9 |
| **27** |  | > 1000 |
| **28** (QP5038) |  | 3.8 ± 0.7 |
| **29** |  | > 1000 |

^a^Results are mean values of three independent experiments or mean ± SD unless specified otherwise.

**2. Supplementary figures**

**Fig. S1** Representative reported QPCT inhibitors.


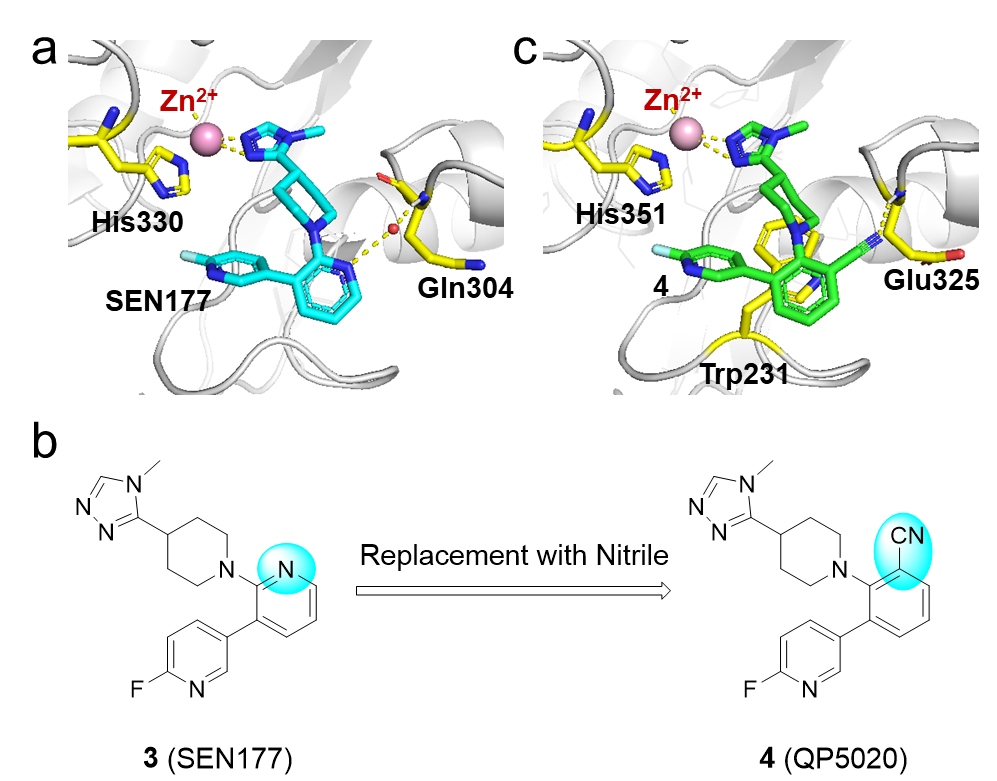


**Fig. S2** **a.** Binding model of SEN177 (cyan) with QPCT (PDB: 6GBX). **b.** Strategy to rationally design **QP5020**. **c**. Predicted binding model of **QP5020** (green) in the catalytically active region of QPCTL (PDB: 3PB7).

**Fig. S3** Fluorescent assay determination of IC_50_ values of **QP5038** against QPCTL and QPCT. The presented data is mean ± SD from three independent experiments.

**Fig. S4** FACS analysis of the interaction between mouse SIRPα and CD47 in B16F10 cells. The data were normalized to DMSO control. Shown is a mean ± SD from three independent experiments. The data was analyzed by Flowjo software and statistically significant differences were determined by one-way ANOVA, ∗∗∗∗ p <0.0001.


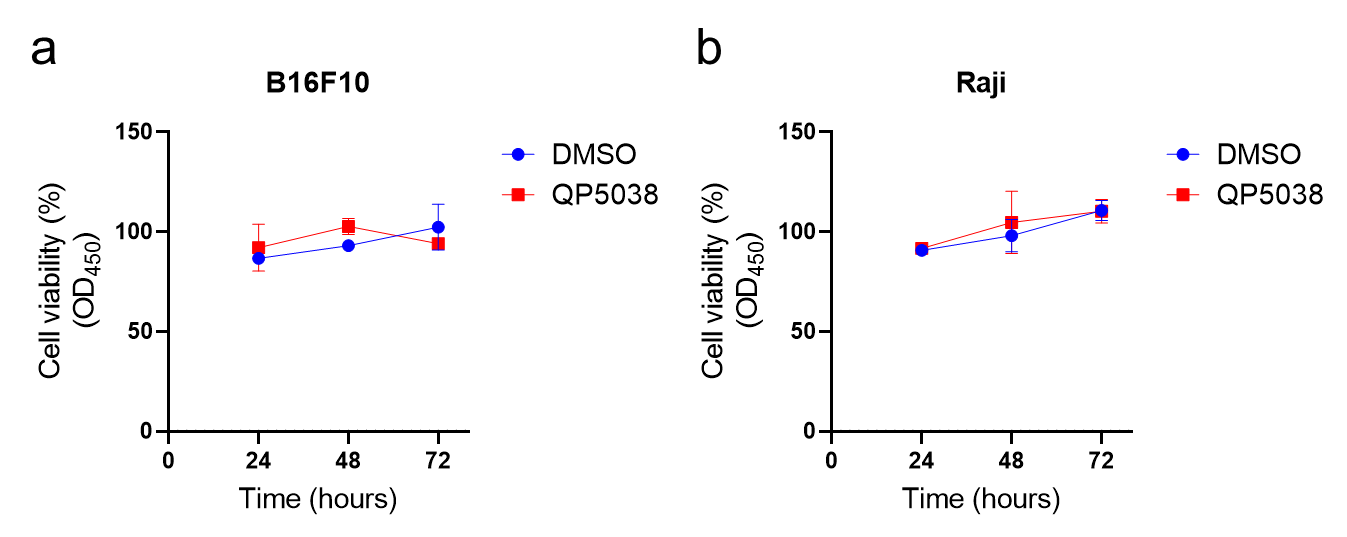


**Fig. S5** **a** and **b**. Cell viability evaluation in Raji and B16F10 cells. The present is a representative, and three rounds of assaying were performed in triplicates.


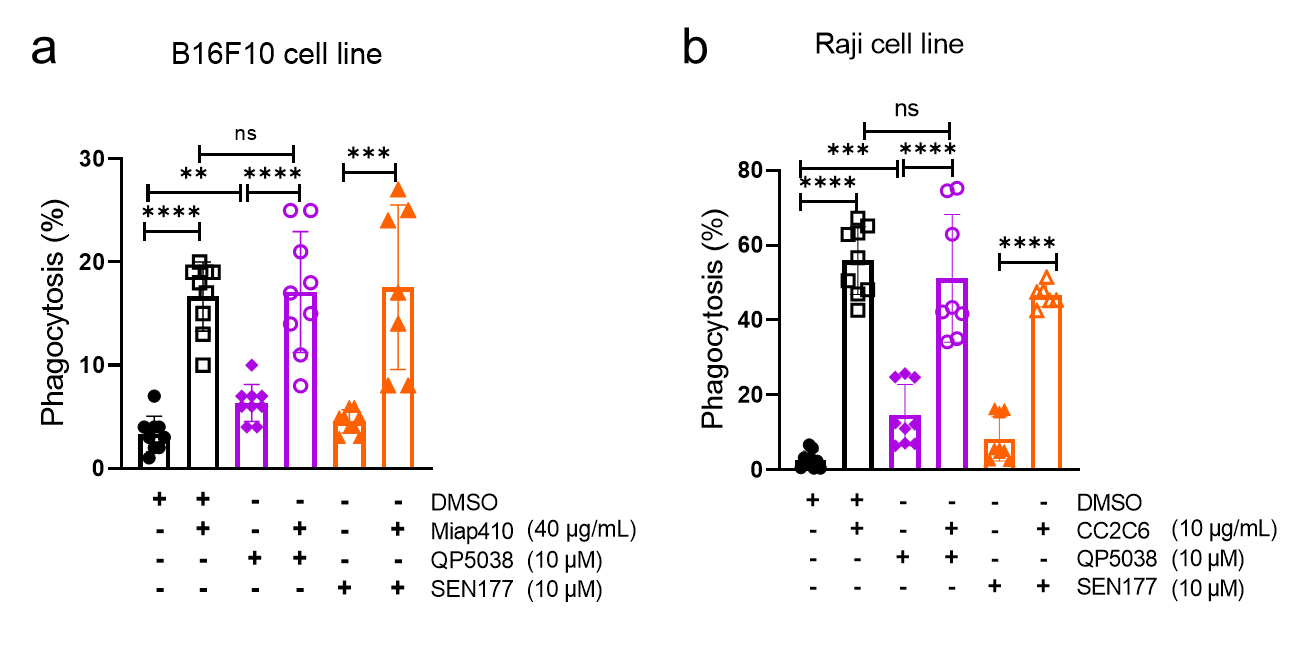


**Fig. S6 a**. Phagocytosis of control-treated (DMSO) (-) or QPCTL inhibitors-treated (+) B16F10 cells in the presence or absence of anti-CD47 antibody Miap410 by mouse macrophages following treatment with 10 μM inhibitors for 48 hours. Phagocytosis was determined by the number of the CFSE ^+^ labelled F4/80^+^ macrophages vs the total tumor cells. **b**. Phagocytosis of control-treated (DMSO) (-) or QPCTL inhibitors-treated (+) Raji cells in the presence or absence of anti-CD47 antibody CC2C6 by mouse macrophages following treatment with 10 μM inhibitors for 48 hours. The presented data is mean ± SD of three independent experiments. Statistically significant differences were determined by unpaired two-tailed *t*-test, ∗∗ p < 0.01, ∗∗∗ p < 0.001, ∗∗∗∗ p < 0.0001, ns, not significant.


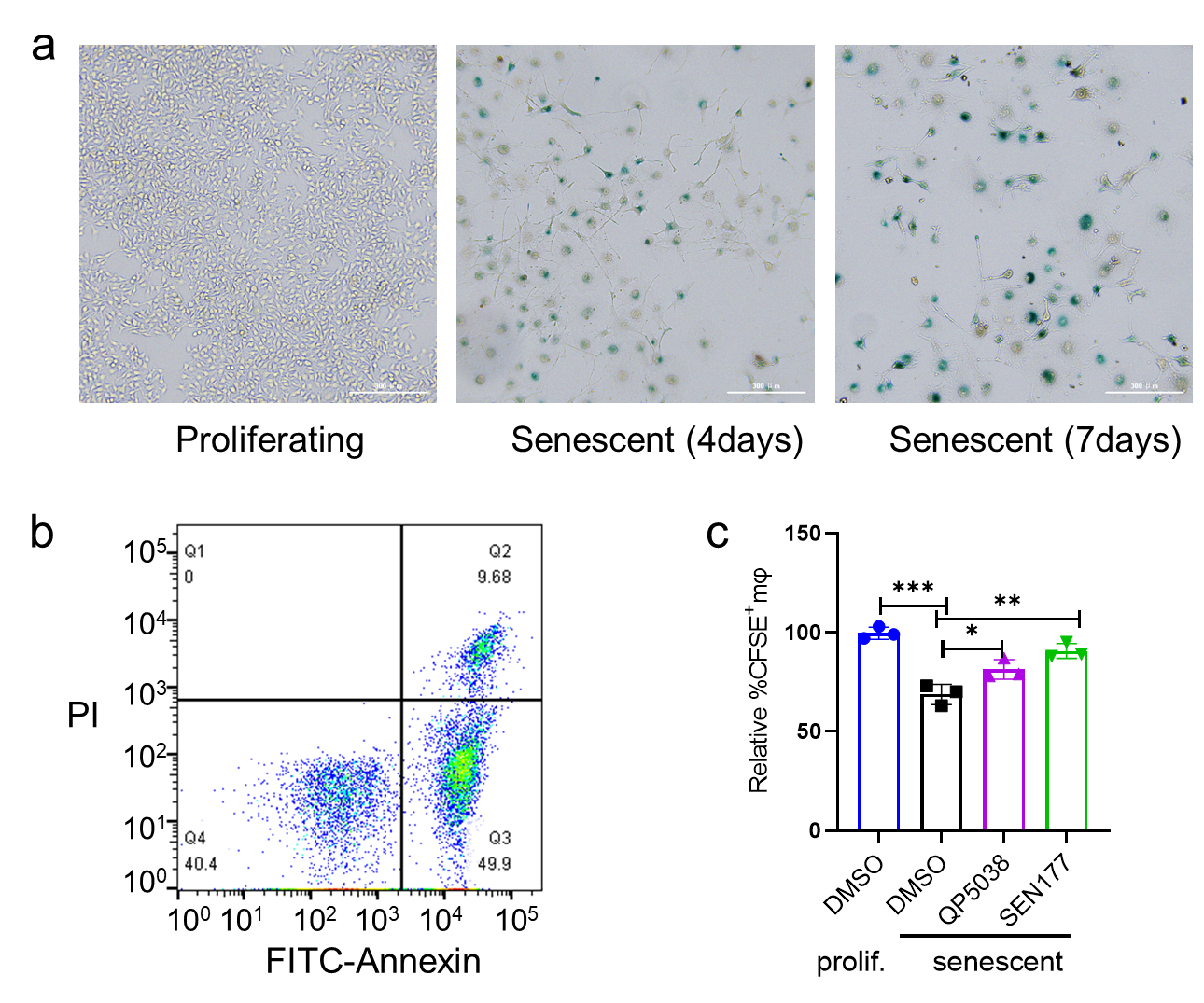


**Fig. S7** **a.** Senescence induction by exposure to 10 μM cisplatin and senescent confirmation by β-gal staining kit. **b**. Apoptotic confirmation of Jurkat cells by flow cytometry. **c**. Efferocytosis in the proliferative (prolif.) and senescent cells (senescent) followed by **QP5038** or SEN177 treatment for 4 days. Data are representative of three independent experiments. Statistically significant differences were determined by one-way ANOVA, ∗ p < 0.05, ∗∗ p < 0.01, ∗∗∗ p < 0.001.

**
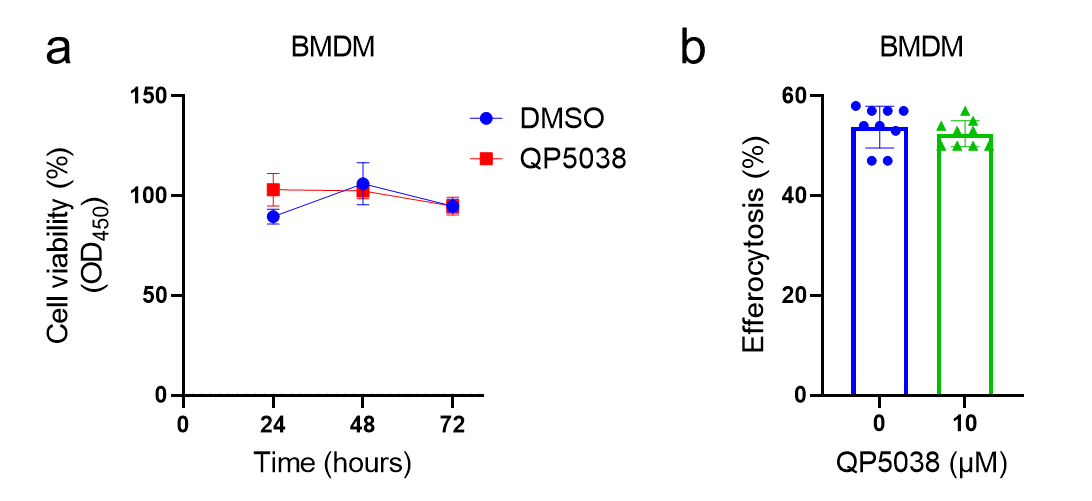
**

**Fig. S8** **a**. Cell viability evaluation in BMDM cells. The present is a representative, and three rounds of assaying were performed in triplicates. **b**. Phagocytosis ability of BMDM to apoptotic Jurkat cells. The presented data is mean ± SD of three independent experiments.


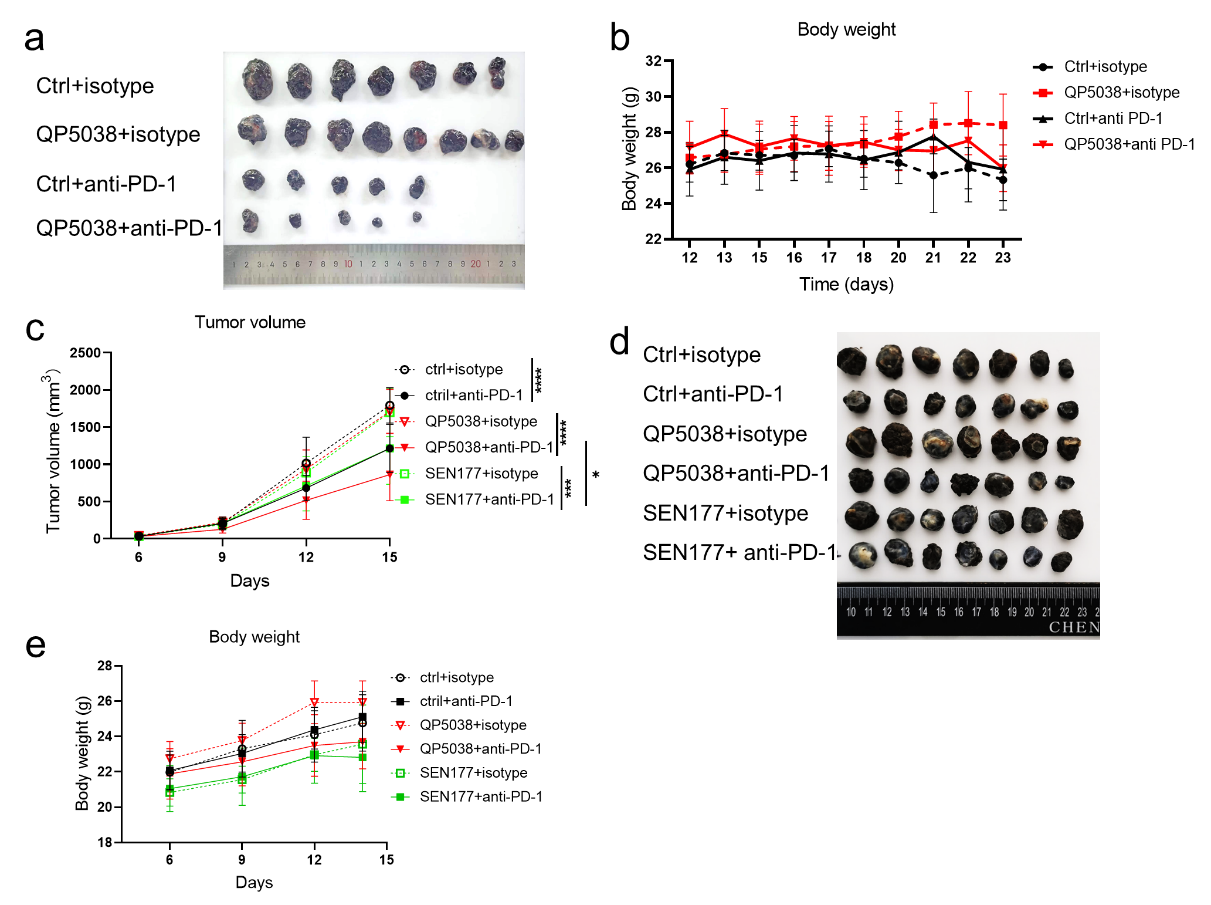


**Fig. S9 a**. Anti-tumor efficacy of **QP5038** and tumor samples when mice are sacrificed. **b**. Body weight changes of mice during treatment. The data were presented as the mean ± SD. **c** and **d**. Anti-tumor efficacy of **QP5038** and SEN177 with once daily intraperitoneally dosing at 25 mg/kg in the presence or absence of the anti-PD-1 antibody. Statistically significant differences were determined by one-way ANOVA, ∗ p < 0.05, ∗∗ p < 0.005, ∗∗∗ p < 0.001, ∗∗∗∗ p < 0.0001. **e**. Body weight changes of mice during treatment. The data were presented as the mean ± SD.


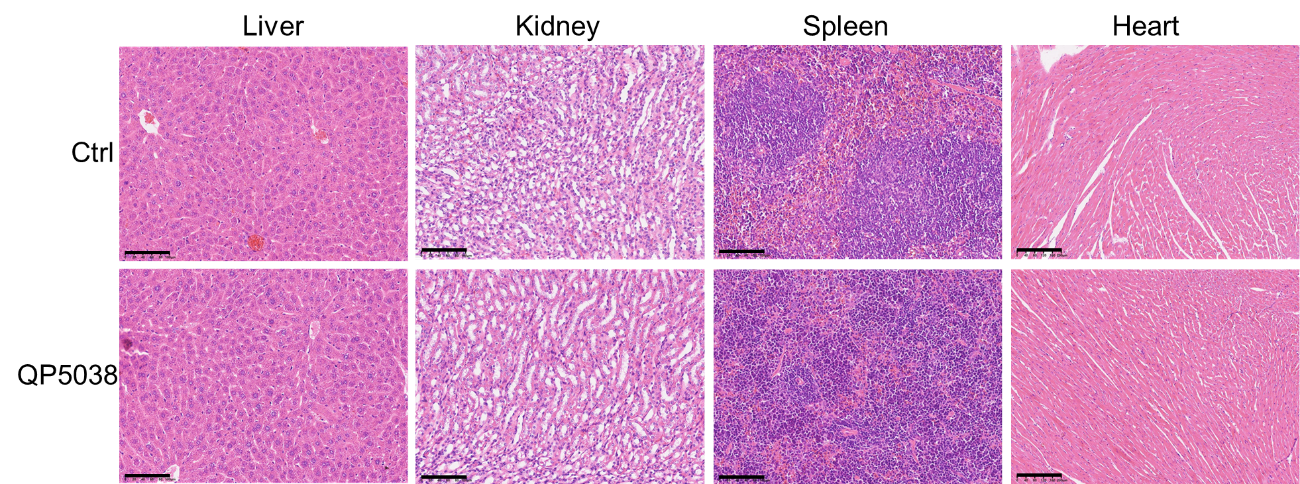


**Fig. S10** Histological examination of C57B/L6 mice by hematoxylin and eosin (HE) staining after treatment with **QP5038 (**25 mg/kg, daily**)** or control vehicle (Ctrl) for 15 days. The present data is a representative image of three mouse. The scale bar is 100 μm.


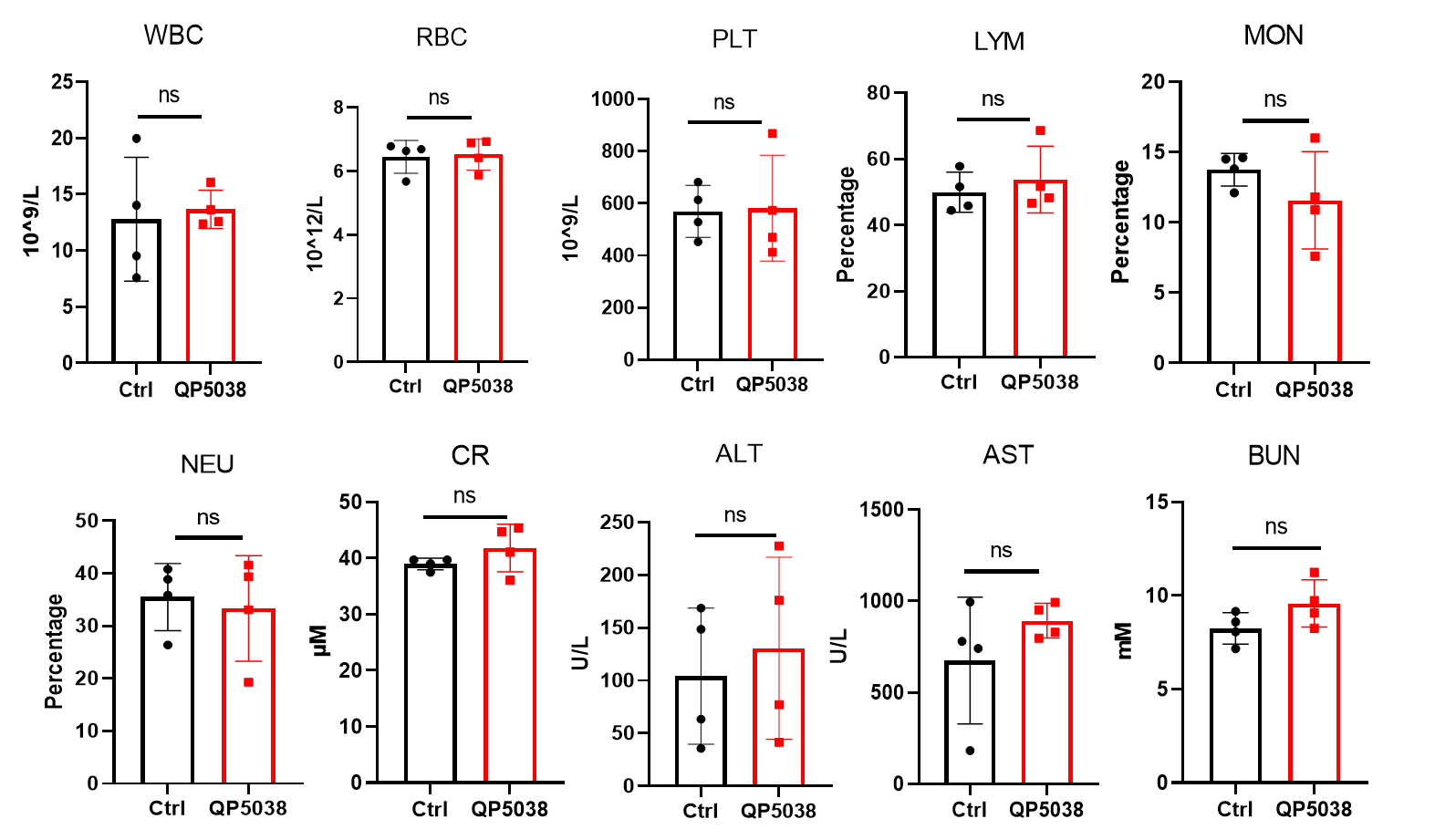


**Fig. S11** Blood routine and blood serum biochemistry examination of mice after treatment with **QP5038** and control vehicle (Ctrl). WBC: whole blood cell, RBC: red blood cell, PLT: blood platelet, LYM: lymphocyte, MON: monocyte, NEU: neutrophil. CR: creatinine, ALT: alanine aminotransferase, AST: aspartate aminotransferase, BUN: blood urea nitrogen. Statistically significant differences were determined by unpaired two-tailed *t*-test, ns, not significant.


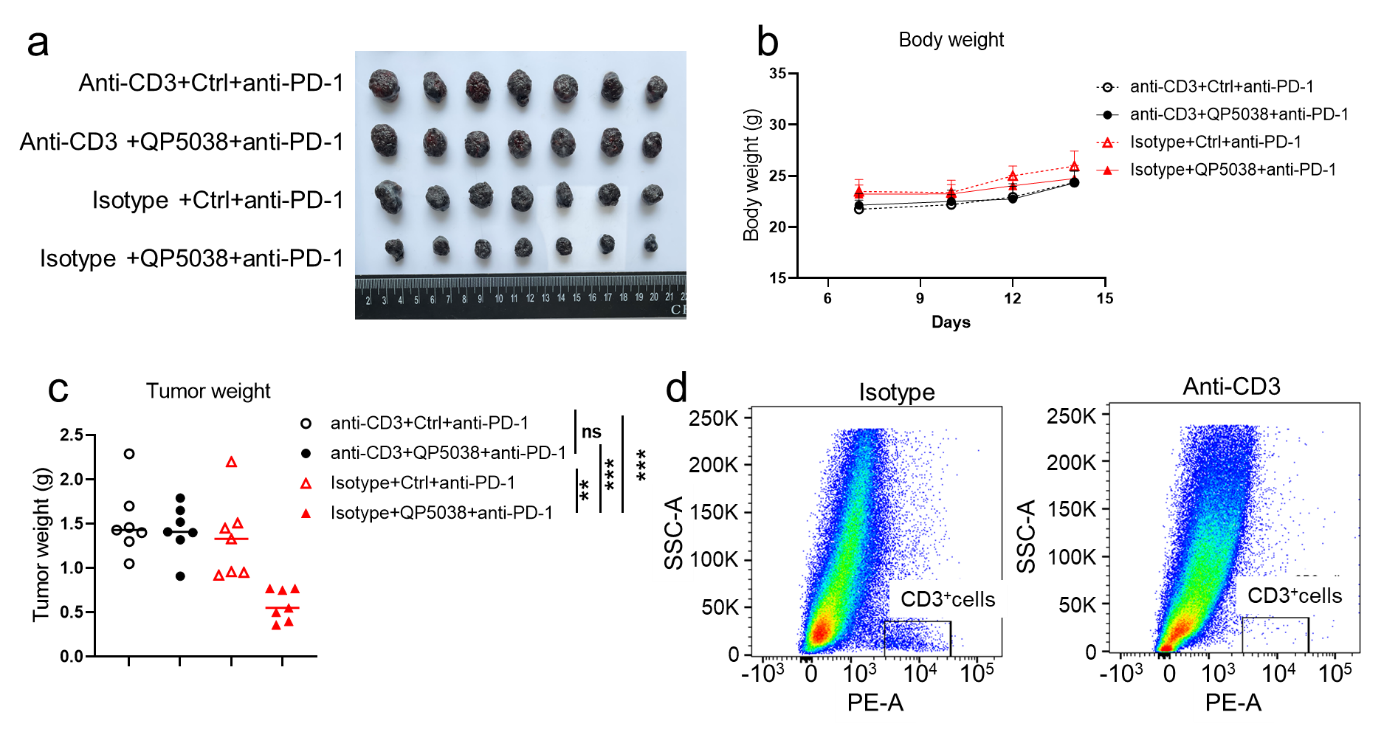


**Fig. S12** **a**. Anti-tumor efficacy of **QP5038** with once daily dosing at 25 mg/kg synergized with anti-PD-1 antibody in the presence or absence of CD3 antibody. **b**. Body weight changes of mice during treatment. The data were presented as the mean ± SD. **c**. Quantification of xenografted tumor weight when mice are sacrificed. The data were presented as the mean ± SD and statistically significant differences were determined by one-way ANOVA, ∗∗ p < 0.005, ∗∗∗ p < 0.001. **d**. Flow cytometry for T cell depletion confirmation.

3. Materials and Methods.

General methods for chemistry

All reagents and anhydrous solvents were obtained from commercial sources without further purification. PQ912 was commercially bought from Topscience Co. Ltd. (Cat. # T22403) and SEN177 was synthesized according to reported methods ^1^. Synthetic reactions were monitored by thin-layer chromatography (TLC) on silica gel F254 plates and visualized by irradiation with UV light at 254 and 366 nm. Final products and reaction intermediates were purified by flash column chromatography or high performance liquid chromatography (HPLC). Proton nuclear magnetic resonance (^1^H NMR) data were recorded on a Bruker Advance 400 or 500M NMR spectrometer or Varian Mercury 400M NMR spectrometer, while ^13^C NMR data was obtained on a Bruker Advance 400 or 500M NMR spectrometer. The chemical shifts were reported in parts per million (ppm) downfield from tetramethylsilane (TMS). The format (*δ*) chemical shift (multiplicity, *J* values in Hz, integration) was used with the following abbreviations: s = singlet, d = doublet, t = triplet, q = quartet, m = multiplet, brs = broad singlet. Low-resolution mass spectra (MS) were recorded on a Thermo Fisher Finnigan LTQ or Agilent G6140 TOF LC/MS spectrometer. The purity of all final compounds was confirmed by UPLC analysis (10% to 100% MeCN in H_2_O containing 0.1% TFA over 10 min).

Detailed synthesis methods

Syntheses of compounds **4**-**25** are outlined in Scheme 1. Commercially available 1-((benzyloxy)carbonyl)piperidine-4-carboxylic acid (**30**) and *N*-methyl-thiosemicarbazide (**31**) were cyclized to generate intermediate **32**, which was subsequently converted to intermediate **33** by removal of the thiol group. Deprotection of compound **33** gave the primary amine **34**, which underwent a S_N_Ar reaction with commercially available compound **35** to produce the key intermediate **36**. Then, the final compounds **4-25** were obtained through Suzuki−Miyaura coupling between intermediate **36** and various boronic acids or boronic acid esters.

Syntheses of compounds **26** and **27** are outlined in Scheme 2. First, Commercially available t*ert*-butyl 4-(hydrazinecarbonyl)piperidine-1-carboxylate (**37**) was cyclized to generate intermediates **38a-b**, which were then converted to **39a-b** by removing the Boc protecting group. **39a-b** were then used to substitute 3-bromo-2-fluorobenzonitrile (**35**) and generate **40a-b**. Finally, compounds **26** and **27** were produced by Suzuki-Miyaura coupling with compound **40a-b** and **41**.

Syntheses of compounds **28** and **29** are outlined in Scheme 3. The starting materials **41a-b** were synthesized using previously reported procedures. Subsequently, **41a-b** were reacted with 3-bromo-2-fluorobenzonitrile (**35**) to afford **42a-b**. Intermediate **42a** was coupled with compound **41** via Suzuki-Miyaura reaction to give final compound **28**. Meanwhile, intermediate **42b** was subjected to Suzuki-Miyaura coupling with compound **41**, followed by deprotection to produce the final compound **29**.

**Scheme 1** Syntheses of compounds **4-25**^a^

^a^Reagents and conditions: (a) CDI, CH_3_CN, 90℃, 18 h; (b) H_2_O_2_, CH_3_COOH, rt, 18 h; (c) 6N HCl, 100℃, 6 h (quantitative); (d) DMF, K_2_CO_3_, 100℃, 18 h; (e) Pd(dppf)Cl_2_, RBpin, Na_2_CO_3_, dioxane/H_2_O (10:1), 90℃, 10 h.

**Scheme 2** Syntheses of compounds **26** and **27**^a^

^a^Reagents and conditions: (a) CH_3_CN, 90℃, 2 h; (b) CH_3_COOH, 90℃, 2 h; (c) 4M HCl in dioxane, MeOH, rt; (d) DMF, K_2_CO_3_, 100℃, 18 h; (e) Pd(dppf)Cl_2_, Na_2_CO_3_, dioxane/H_2_O (10:1), 90℃, 10 h.

**Scheme 3** Syntheses of compounds **28** and **29**^a^

^a^Reagents and conditions: (a) DMF, K_2_CO_3_, 100℃, 18 h; (b) Pd(dppf)Cl_2_, Na_2_CO_3_, dioxane/H_2_O (10:1), 90℃, 10 h; (c) TFA, DCM, rt, 16 h and 7M NH_3_ in MeOH, rt, 3h.

**3-(6-Fluoropyridin-3-yl)-2-(4-(4-methyl-4*H*-1,2,4-triazol-3-yl)piperidin-1-yl)benzonitrile** (**4**). Intermediate **36** (35 mg, 0.10 mmol, 1 eq), Na_2_CO_3_ (21 mg, 0.2 mmol, 2.0 eq) and 4-fluoro-3-pyridyl-boronic acid (34 mg, 0.15 mmol, 1.5 eq) were dissolved in dioxane (2 mL) and H_2_O (0.2 mL), and the resulting mixture was degassed under N_2_ flux. Pd_3_(dba)_2_ (7 mg, 0.01 mmol, 0.1 eq) were added, and the reaction was heated at 90 ℃ for 10 hours under inert atmosphere. The mixture was allowed to cool to room temperature, and then was diluted with H_2_O and extracted with ethyl acetate. The organic layer was concentrated, and 20 mg of the white solid compound was obtained (yield, 55.2%) as trifluoroacetate salt after purification by prep-HPLC and lyophilization. ^1^H NMR (400MHz, MeOD-*d_4_*) δ 8.98 (s, 1H), 8.28 (d, *J* = 2.4 Hz, 1H), 8.05 (td, *J* = 8.4, 2.4 Hz, 1H), 7.75 (dd, *J* = 7.6, 1.6 Hz, 1H), 7.58 (dd, *J* = 8.0, 2.0 Hz, 1H), 7.35 (t, *J* = 7.6 Hz, 1H), 7.23 (dd, *J* = 8.4, 2.4 Hz, 1H), 3.86 (s, 3H), 3.29 (brs, 2H), 3.18-3.09 (m,3H), 1.98-1.94 (m, 2H), 1.85-1.81 (m, 2H). ^13^C NMR (125MHz, MeOD-*d4*) δ 164.4 (d, *J* = 238.1 Hz), 159.2, 154.2, 148.5 (d, *J* = 14.1 Hz), 145.8, 144.1 (d, *J* = 8.1 Hz), 137.6, 136.9, 135.8, 135.1 (d, *J* = 3.4 Hz), 126.0, 119.4, 111.2, 110.5 (d, *J* = 37.0 Hz), 52.4, 33.0, 32.7, 30.7. HRMS (ESI) calcd. for C_20_H_19_FN_6_ [M + H]^+^ 363.1733, found: 363.1728. HPLC: t_R_ = 6.29 min, purity: 97.3%.

**2-(4-(4-Methyl-4*H*-1,2,4-triazol-3-yl)piperidin-1-yl)-3-(pyridin-3-yl)benzonitrile** (**5**). Compound **5** was synthesized using the similar procedure as compound **4**. ^1^H NMR (400MHz, MeOD-*d_4_*) δ 9.07 (s, 1H), 8.98 (s, 1H), 8.82 (d, *J* = 5.6 Hz, 1H), 8.53 (d, *J* = 7.6 Hz, 1H), 8.05 (m, 1H), 7.82 (dd, *J* = 7.6, 1.2. Hz, 1H), 7.69 (d, *J* = 7.6 Hz, 1H), 7.43 (t, *J* = 7.8 Hz, 1H), 3.85 (s, 3H), 3.26 (s, 1H), 3.15 (m, 1H), 1.95 (d, *J* =11.6 Hz, 2H), 1.72 (s, 2H); LC-MS (ESI) calcd. for C_20_H_20_N_6_ [M + H]^+^ 345.2, found 345.3. HPLC: t_R_ = 4.13 min, purity: 95.9%.

**2-(4-(4-Methyl-4*H*-1,2,4-triazol-3-yl)piperidin-1-yl)-[1,1'-biphenyl]-3-carbonitrile** (**6**). Compound **6** was synthesized using the similar procedure as compound **4**. ^1^H NMR (400MHz, MeOD-*d_4_*) δ 9.11 (s, 1H), 7.65 (dd, *J* = 1.6, 7.6 Hz, 1H), 7.54-7.43 (m, 3H), 7.40 (d, *J* = 7.2 Hz, 1H), 7.36 (d, *J* = 7.2 Hz, 2H), 7.24 (t, *J* = 7.6 Hz, 1H), 3.85 (s, 3H), 3.28 (s, 1H), 3.15-3.02 (m, 1H), 3.01-2.91 (m, 2H), 1.94-1.87 (m, 4H); LC-MS (ESI) calcd. for C_21_H_21_N_5_ [M + H]^+^ 344.2, found 344.4. HPLC: t_R_ = 7.15 min, purity: 95.9%.

**2-(4-(4-Methyl-4*H*-1,2,4-triazol-3-yl)piperidin-1-yl)-3-(pyridin-4-yl)benzonitrile** (**7**). Compound **7** was synthesized using the similar procedure as compound **4**. ^1^H NMR (400MHz, MeOD-*d_4_*) δ 8.91-8.89 (m, 3H), 8.06 (d, *J* = 6.0 Hz, 2H), 7.85 (dd, *J* =7.6, 1.6 Hz, 1H), 7.68 (dd, *J* =7.6, 1.6 Hz, 1H), 7.42 (t, *J* = 7.8 Hz, 1H), 3.83 (s, 3H), 3.18-3.10 (m, 1H), 1.96-1.93 (m, 2H), 1.82-1.80 (br, 2H); LC-MS (ESI) calcd. for C_20_H_20_N_6_ [M + H]^+^ 344.4, found 345.1. HPLC: t_R_ = 4.17 min, purity: 99.9%.

**2-(4-(4-Methyl-4*H*-1,2,4-triazol-3-yl)piperidin-1-yl)benzonitrile** (**8**). Compound **8** was synthesized using the similar procedure as compound **4**. ^1^H NMR (400MHz, MeOD-*d_4_*) δ 9.08 (s, 1H), 7.65-7.57 (m, 2H), 7.21 (d, *J* = 8.4 Hz, 1H), 7.11 (t, *J* = 7.6 Hz, 1H), 3.93 (s, 3H), 3.68 (d, *J* = 12.4 Hz, 2H), 3.33-3.26 (m, 2H), 3.08-3.00 (m, 2H), 2.24-2.03 (m, 4H); LC-MS (ESI) calcd. for C_15_H_17_N_5_ [M + H]^+^ 268.1, found 268.3. HPLC: t_R_ = 5.44 min, purity: 95.8%.

**2-(4-(4-Methyl-4*H*-1,2,4-triazol-3-yl)piperidin-1-yl)-3-(pyrimidin-5-yl)benzonitrile** (**9**). Compound **9** was synthesized using the similar procedure as compound **4**. ^1^H NMR (400MHz, MeOD-*d_4_*) δ 9.18 (d, *J* = 2.8 Hz, 2H), 8.92 (s, 2H), 7.78 (dd, *J* = 8.0, 1.6 Hz, 1H), 7.66 (dd, *J* = 8.0, 1.6 Hz, 1H), 7.40 (t, *J* = 7.6 Hz, 1H), 3.89 (s, 3H), 3.35-3.15 (m, 5H), 2.04-1.94 (m, 2H), 1.74 (brs, 2H); LC-MS (ESI) calcd. for C_19_H_19_N_7_ [M + H]^+^ 346.2, found 346.3. HPLC: t_R_ = 5.28 min, purity: 97.9%.

**2-(4-(4-Methyl-4*H*-1,2,4-triazol-3-yl)piperidin-1-yl)-3-(6-(trifluoromethyl) pyridin-3-yl)benzonitrile** (**10**). Compound **10** was synthesized using the similar procedure as compound **4**. ^1^H NMR (400MHz, MeOD-*d_4_*) δ 9.21 (s, 1H), 8.80 (d, *J* = 1.2 Hz, 1H), 8.12 (dd, *J* = 8.0, 2.0 Hz, 1H), 7.96 (d, *J* = 8.0 Hz, 1H), 7.77 (dd, *J* = 7.6, 1.6 Hz, 1H), 7.61 (dd, *J* = 7.6, 1.6 Hz, 1H), 7.37 (t, *J* = 7.6 Hz, 1H), 3.89 (s, 3H), 3.32-3.28 (m, 2H), 3.24-3.13 (m, 3H), 1.99-1.94 (m, 2H), 1.82-1.78 (m, 2H); LC-MS (ESI) calcd. for C_21_H_19_F_3_N_6_ [M + H]^+^ 413.2, found 413.4. HPLC: t_R_ = 6.95 min, purity: 91.6%.

**5-(3-Cyano-2-(4-(4-methyl-4*H*-1,2,4-triazol-3-yl)piperidin-1-yl)phenyl)picolinonitrile** (**11**)**.** Compound **11** was synthesized using the similar procedure as compound **4**. ^1^H NMR (400MHz, MeOD-*d_4_*) δ 9.23 (s, 1H), 8.82 (d, *J* = 1.2 Hz, 1H), 8.08 (dd, *J* = 8.0, 2.0 Hz, 1H), 8.01 (d, *J* = 8.0 Hz, 1H), 7.78 (dd, *J* = 8.0, 1.6 Hz, 1H), 7.63 (dd, *J* = 7.8, 2.0 Hz, 1H), 7.40 (t, *J* = 7.8 Hz, 1H), 3.92 (s, 3H), 3.30-3.25 (m, 2H), 3.25-3.21 (m, 1H), 3.21-3.10 (m, 2H), 2.01-1.96 (m, 2H), 1.87-1.65 (m, 2H). ESI-MS calcd. for C_21_H_19_N_7_ [M + Na]^+^ 392.2, found: 392.4. HPLC: t_R_ = 6.19 min, purity: 99.9%.

**3-(6-Aminopyridin-3-yl)-2-(4-(4-methyl-4*H*-1,2,4-triazol-3-yl)piperidin-1-yl)benzonitrile** (**12**). Compound **12** was synthesized using the similar procedure as compound **4**. ^1^H NMR (400MHz, MeOD-*d_4_*) δ 9.21 (s, 1H), 8.03 (d, *J* = 9.2 Hz, 1H), 7.97-7.89 (m, 1H), 7.72 (dd, *J* = 7.6, 1.2 Hz, 1H), 7.58 (d, *J* = 7.6 Hz, 1H), 7.33 (t, *J* = 7.6 Hz, 1H), 7.12 (d, *J* = 9.2 Hz, 1H), 3.90 (s, 3H), 3.34-3.19 (m, 5H), 2.05-2.00 (m, 2H), 1.97-1.73 (m, 2H); LC-MS (ESI) calcd. for C_20_H_21_N_7_ [M + H]^+^ 360.2, found 360.3. HPLC: t_R_ = 4.83 min, purity: 99.9%.

**3-(6-Methoxypyridin-3-yl)-2-(4-(4-methyl-4*H*-1,2,4-triazol-3-yl)piperidin-1-yl) benzonitrile** (**13**). Compound **13** was synthesized using the similar procedure as compound **4**. ^1^H NMR (400MHz, MeOD-*d_4_*) δ 9.19 (s, 1H), 8.17 (d, *J* = 2.0 Hz, 1H), 7.79 (dd, *J* =8.4, 1.6 Hz, 1H), 7.68 (dd, *J* = 7.6, 1.2 Hz, 1H), 7.51 (dd, *J* = 7.8 ,1.6 Hz, 1H), 7.29 (t, *J* = 7.8 Hz, 1H), 6.96 (d, *J* = 8.6 Hz, 1H), 3.97 (s, 3H), 3.89 (s, 3H), 3.28 (s, 1H), 3.22-3.06 (m, 3H), 1.97 (d, *J* = 10.8 Hz, 2H), 1.92-1.80 (m, 2H); LC-MS (ESI) calcd. for C_21_H_22_N_6_O [M + H]^+^ 375.2, found 375.4. HPLC: t_R_ = 6.38 min, purity: 98.0%.

**3-(5-Fluoropyridin-3-yl)-2-(4-(4-methyl-4*H*-1,2,4-triazol-3-yl)piperidin-1-yl)benzonitrile** (**14**). Compound **14** was synthesized using the similar procedure as compound **4**. ^1^H NMR (400MHz, MeOD-*d_4_*) δ 9.23 (s, 1H), 8.55-8.52 (m, 2H), 7.81 (d, *J* = 9.2 Hz, 1H), 7.75 (dd, *J* = 7.6, 1.2 Hz, 1H), 7.59 (d, *J* = 7.6 Hz, 1H), 7.35 (t, *J* = 7.6 Hz, 1H), 3.90 (s, 3H), 3.34-3.06 (m, 5H), 2.03-1.93 (m, 2H), 1.91-1.69 (m, 2H); LC-MS (ESI) calcd. for C_20_H_19_FN_6_ [M + Na]^+^ 385.2, found 385.3. HPLC: t_R_ = 5.93 min, purity: 99.9%.

**5-(3-Cyano-2-(4-(4-methyl-4*H*-1,2,4-triazol-3-yl)piperidin-1-yl)phenyl)nicotinonitrile** (**15**). Compound **15** was synthesized using the similar procedure as compound **4**. ^1^H NMR (400MHz, MeOD-*d_4_*) δ 9.21 (s, 1H), 8.94-8.89 (m, 2H), 8.31 (s, 1H), 7.77 (d, *J* = 7.6 Hz, 1H), 7.62 (d, *J* = 7.6 Hz, 1H), 7.38 (t, *J* = 7.6 Hz, 1H), 3.89 (s, 3H), 3.38-3.10 (m, 5H), 2.02-1.90 (m, 2H), 1.89-1.52 (m, 2H); LC-MS (ESI) calcd. for C_21_H_19_N_7_ [M + H]^+^ 370.2, found 370.4. HPLC: t_R_ = 6.04 min, purity: 99.8%.

**3-(4-Methoxypyridin-3-yl)-2-(4-(4-methyl-4*H*-1,2,4-triazol-3-yl)piperidin-1-yl)benzonitrile** (**16**). Compound **16** was synthesized using the similar procedure as compound **4**. ^1^H NMR (400MHz, MeOD-*d_4_*) δ 9.14 (s, 1H), 7.76 (d, *J* = 6.8 Hz, 1H), 7.73 (dd, *J* = 7.6, 1.6 Hz, 1H), 7.52 (dd, *J* = 7.6, 1.6 Hz, 1H), 7.28 (t, *J* = 7.6 Hz, 1H), 6.57 (d, *J* = 1.6 Hz, 1H), 6.47 (dd, *J* = 6.8, 2.0 Hz, 1H), 3.90 (s, 3H), 3.63 (s, 3H), 3.41-3.36 (m, 2H), 3.26-3.18 (m, 3H), 2.04-1.93 (m, 4H); LC-MS (ESI) calcd. for C_21_H_22_N_6_O [M + H]^+^ 375.2, found 375.3. HPLC: t_R_ = 5.46 min, purity: 98.4%.

**2-(4-(4-Methyl-4*H*-1,2,4-triazol-3-yl)piperidin-1-yl)-3-(4-(trifluoromethyl)pyridin-3-yl)benzonitrile** (**17**). Compound **17** was synthesized using the similar procedure as compound **4**. ^1^H NMR (400MHz, MeOD-*d_4_*) δ 9.14 (s, 1H), 8.86 (d, *J* = 5.2 Hz, 1H), 8.68 (s, 1H), 7.88 (d, *J* = 5.2 Hz, 1H), 7.81 (dd, *J* = 7.6, 1.6 Hz, 1H), 7.56 (d, *J* = 7.6 Hz, 1H), 7.38 (t, *J* = 7.6 Hz, 1H), 3.84 (s, 3H), 3.29-3.21 (m, 3H), 3.15-3.06 (m, 2H), 1.93-1.89 (m, 2H), 1.57-1.53 (m, 2H); LC-MS (ESI) calcd. for C_21_H_19_F_3_N_6_ [M + H]^+^ 413.2, found 413.1. HPLC: t_R_ = 6.70 min, purity: 96.7%.

**2-(4-(4-Methyl-4*H*-1,2,4-triazol-3-yl)piperidin-1-yl)-3-(4-methylpyridin-3-yl)benzonitrile** (**18**). Compound **18** was synthesized using the similar procedure as compound **4**. ^1^H NMR (400MHz, MeOD-*d_4_*) δ 9.00 (s, 1H), 8.79 (s, 1H), 8.74 (d, *J* = 6.0 Hz, 1H), 8.04 (d, *J* = 6.0 Hz, 1H), 7.85 (dd, *J* = 7.6, 1.2 Hz, 1H), 7.60 (dd, *J* = 7.6, 1.2 Hz, 1H), 7.43 (t, *J* = 7.6 Hz, 1H), 3.82 (s, 3H), 3.44-3.33 (m, 1H), 3.24 (s, 3H), 3.10 (m, 1H), 2.48 (s, 3H), 1.90 (t, *J* = 10.4 Hz, 2H), 1.55-1.43 (m, 2H); LC-MS (ESI) calcd. for C_21_H_22_N_6_ [M + H]^+^ 359.2, found 359.4. HPLC: t_R_ = 4.64 min, purity: 94.6 %.

**3-(4-Chloropyridin-3-yl)-2-(4-(4-methyl-4*H*-1,2,4-triazol-3-yl)piperidin-1-yl)benzonitrile** (**19**). Compound **19** was synthesized using the similar procedure as compound **4**. ^1^H NMR (400MHz, MeOD-*d_4_*) δ 9.23 (s, 1H), 8.67-8.63 (m, 2H), 7.87 (d, *J* = 5.6 Hz, 1H), 7.81 (d, *J* = 7.6 Hz, 1H), 7.55 (d, *J* = 7.6 Hz, 1H), 7.38 (t, *J* = 7.6 Hz, 1H), 3.87 (s, 3H), 3.28-3.22 (m, 2H), 3.19-3.11 (m, 3H), 1.98-1.89 (m, 2H), 1.69-1.54 (m, 2H); LC-MS (ESI) calcd. for C_20_H_19_ClN_6_ [M + H]^+^ 379.1, found 379.5. HPLC: t_R_ = 5.70 min, purity: 99.6%.

**2-(4-(4-Methyl-4*H*-1,2,4-triazol-3-yl)piperidin-1-yl)-3-(6-oxo-1,6-dihydropyridin-3-yl)benzonitrile** (**20**). Compound **20** was synthesized using the similar procedure as compound **4**. ^1^H NMR (400MHz, MeOD-*d_4_*) δ 9.12 (s, 1H), 7.72 (d, *J* = 8.8 Hz, 1H), 7.67 (dd, *J* = 7.6, 1.4 Hz, 1H), 7.54-7.52 (m, 2H), 7.29 (t, *J* = 7.6 Hz, 1H), 6.65 (d, *J* = 9.2 Hz, 1H), 3.89 (s, 3H), 3.39-3.33 (m, 4H), 3.24-3.16 (m, 1H), 2.04-1.90 (m, 4H); LC-MS calcd. for C_20_H_20_N_6_O [M + H]^+^ 361.2, found 361.1. HPLC: t_R_ = 5.26 min, purity: 99.5%.

**2-(4-(4-Methyl-4*H*-1,2,4-triazol-3-yl)piperidin-1-yl)-3-(1-methyl-6-oxo-1,6-dihydropyridin-3-yl)benzonitrile** (**21**). Compound **21** was synthesized using the similar procedure as compound **4**. ^1^H NMR (400MHz, MeOD-*d_4_*) δ 9.21 (s, 1H), 7.78 (d, *J* = 2.2 Hz, 1H), 7.65 (dd, *J* = 7.6, 1.2 Hz, 2H), 7.54 (dd, *J* = 7.6, 1.2 Hz, 1H), 7.29 (t, *J* = 7.6 Hz, 1H), 6.65 (d, *J* = 9.4 Hz, 1H), 3.90 (s, 3H), 3.65 (s, 3H), 3.37-3.18 (m, 4H), 2.02 (d, *J* = 11.2 Hz, 2H), 1.96-1.84 (m, 2H); LC-MS (ESI) calcd. for C_21_H_22_N_6_O [M + H]^+^ 375.2, found 375.4. HPLC: t_R_ = 5.53 min, purity: 93.6%.

**2-(4-(4-Methyl-4*H*-1,2,4-triazol-3-yl)piperidin-1-yl)-3-(2-oxo-1,2-dihydropyridin-4-yl)benzonitrile** (**22**). Compound **22** was synthesized using the similar procedure as compound **4**. ^1^H NMR (400MHz, MeOD-*d_4_*) δ 9.20 (s, 1H), 7.74 (dd, *J* = 7.6, 1.2 Hz, 1H), 7.58 (d, *J* = 6.8 Hz, 1H), 7.54 (d, *J* = 7.6 Hz, 1H), 7.29 (t, *J* = 7.6 Hz, 1H), 6.58 (s, 1H), 6.52 (d, *J* = 6.8 Hz, 1H), 3.92 (s, 3H), 3.43-3.37 (m, 2H), 3.28-3.21 (m, 3H), 2.06-1.93 (m, 4H); LC-MS (ESI) calcd. for C_20_H_20_N_6_O [M + H]^+^ 361.2, found 361.2. HPLC: t_R_ = 5.26 min, purity: 95.1%.

**3-(1-Methyl-2-oxo-1,2-dihydropyridin-4-yl)-2-(4-(4-methyl-4*H*-1,2,4-triazol-3-yl)piperidin-1-yl)benzonitrile** (**23**). Compound **23** was synthesized using the similar procedure as compound **4**. ^1^H NMR (400MHz, MeOD-*d4*) δ 9.13 (s, 1H), 8.83 (dd, *J* = 6.8, 1.2 Hz, 1H), 8.69 (d, *J* = 1.2 Hz, 1H), 7.82 (dd, *J* = 7.6, 1.6 Hz, 1H), 7.78 (d, *J* = 7.2 Hz, 1H), 7.58 (dd, *J* = 7.8, 1.2 Hz, 1H), 7.39 (t, *J* = 7.8 Hz, 1H), 4.18 (s, 3H), 3.86 (s, 3H), 3.26-3.13 (m, 5H), 2.00-1.94 (m, 2H), 1.63 (brs, 2H); LC-MS (ESI) calcd. for C_21_H_22_N_6_O [M + H]^+^ 375.2, found 375.2. HPLC: t_R_ = 4.71 min, purity: 99.5%.

**3-(Furan-3-yl)-2-(4-(4-methyl-4*H*-1,2,4-triazol-3-yl)piperidin-1-yl)benzonitrile** (**24**). Compound **24** was synthesized using the similar procedure as compound **4**. ^1^H NMR (400MHz, MeOD-*d_4_*) δ 9.06 (s, 1H), 7.86 (s, 1H), 7.63-7.58 (m, 3H), 7.26 (t, *J* = 7.6 Hz, 1H), 6.72 (s, 1H), 3.90 (s, 3H), 3.34-3.32 (m, 4H), 3.25-3.17 (m, 1H), 2.06-2.00 (m, 4H); LC-MS (ESI) calcd. for C_19_H_19_N_5_O [M + H]^+^ 334.2, found 334.2. HPLC: t_R_ = 6.71 min, purity: 97.4%.

**3-(3,5-Dimethylisoxazol-4-yl)-2-(4-(4-methyl-4*H*-1,2,4-triazol-3-yl)piperidin-1-yl)benzonitrile** (**25**). Compound **25** was synthesized using the similar procedure as compound **4**. ^1^H NMR (400MHz, MeOD-*d_4_*) δ 9.07 (s, 1H), 7.72 (dd, *J* = 7.6, 2.0 Hz, 1H), 7.42 (dd, *J* = 7.6, 1.6 Hz, 1H), 7.30 (t, *J* = 7.6 Hz, 1H), 3.87 (s, 3H), 3.39-3.36 (m, 2H), 3.28-3.15 (m, 3H), 2.32 (s, 3H), 2.15 (s, 3H), 2.05-1.94 (m, 2H), 1.90-1.70 (m, 2H); LC-MS (ESI) calcd. for C_20_H_22_N_6_O [M + H]^+^ 363.2, found 363.3. HPLC: t_R_ = 6.33 min, purity: 97.1%.

**2-(4-(4,5-Dimethyl-4*H*-1,2,4-triazol-3-yl)piperidin-1-yl)-3-(6-fluoropyridin-3-yl)benzonitrile** (**26**). Compound **26** was synthesized using the similar procedure as compound **27**. ^1^H NMR (400MHz, MeOD-*d_4_*) δ 8.28 (d, *J* = 2.4 Hz, 1H), 8.04 (td, *J* = 8.0, 2.8 Hz, 1H), 7.75 (dd, *J* = 8.0, 2.0 Hz, 1H), 7.58 (dd, *J* = 7.6, 2.0 Hz, 1H), 7.35 (t, *J* = 7.6 Hz, 1H), 7.22 (dd, *J* = 8.4, 2.8 Hz, 1H), 3.74 (s, 3H), 3.30-3.28 (m, 2H), 3.17-3.07 (m, 3H), 2.62 (s, 3H), 1.97-1.93 (m, 2H), 1.87-1.77 (m, 2H). LC-MS (ESI) calcd. for C_21_H_21_FN_6_ [M + H]^+^ 377.2, found: 377.2. HPLC: t_R_ = 6.45 min, purity: 91.3%.

**3-(6-Fluoropyridin-3-yl)-2-(4-(4-isopropyl-4*H*-1,2,4-triazol-3-yl)piperidin-1-yl)benzonitrile** (**27**). Intermediate **40b** (38 mg, 0.10 mmol, 1 eq), Na_2_CO_3_ (21 mg, 0.2 mmol, 2.0 eq) and 4-fluoro-3-pyridyl-boronic acid (34 mg, 0.15 mmol, 1.5 eq) were dissolved in dioxane (2 mL) and H_2_O (0.2 mL) and the resulting mixture was degassed under N_2_ flux. Pd(dppf)Cl_2_ (7 mg, 0.01 mmol, 0.1 eq) were added, and the reaction was heated at 90 ℃ for 10 hours under inert atmosphere. The mixture was allowed to cool to room temperature and then was diluted with H_2_O and extracted with ethyl acetate. The organic layer was concentrated, and 26 mg of the title compound was obtained (yield, 66.7%) as trifluoroacetate salt after purification by prep-HPLC and lyophilization. ^1^H NMR (400MHz, MeOD-*d_4_*) δ 9.34 (s, 1H), 8.29 (d, *J* = 2.4 Hz, 1H), 8.05 (td, *J* = 8.0, 2.4 Hz, 1H), 7.75 (dd, *J* = 8.0, 1.6 Hz, 1H), 7.58 (dd, *J* = 8.0, 2.0 Hz, 1H), 7.35 (t, *J* = 8.0 Hz, 1H), 7.23 (dd, *J* = 8.4, 2.4 Hz, 1H), 4.80-4.69 (m, 1H), 3.31-3.28 (m, 2H), 3.22-3.13 (m, 3H), 1.96-1.86 (m, 4H), 1.58 (d, *J* = 6.8 Hz, 6H). LC-MS (ESI) calcd. for C_22_H_23_FN_6_ [M + H]^+^ 391.2, found: 391.2. HPLC: t_R_ = 6.63 min, purity: 99.5%.

**3-(6-Fluoropyridin-3-yl)-2-(4-(1-methyl-1*H*-imidazol-5-yl)piperidin-1-yl)benzonitrile** (**28**). Intermediate **42a** (35 mg, 0.10 mmol, 1 eq), Na_2_CO_3_ (21 mg, 0.2 mmol, 2.0 eq) and 4-fluoro-3-pyridyl-boronic acid (34 mg, 0.15 mmol, 1.5 eq) were dissolved in dioxane (2 mL) and H_2_O (0.2 mL) and the resulting mixture was degassed under N_2_ flux. Pd(dppf)Cl_2_ (7 mg, 0.01 mmol, 0.1 eq) were added, and the reaction was heated at 90 ℃ for 10 hours under inert atmosphere. The mixture was allowed to cool to room temperature and then was diluted with H_2_O and extracted with ethyl acetate. The organic layer was concentrated, and 17 mg of the white solid compound was obtained (yield, 46.9%) as trifluoroacetate salt after purification by prep-HPLC and lyophilization. ^1^H NMR (400MHz, MeOD-*d_4_*) δ 8.80 (s, 1H), 8.30 (d, *J* = 2.4 Hz, 1H), 8.05 (td, *J* = 8.0, 2.4 Hz, 1H), 7.74 (dd, *J* = 7.6, 1.6 Hz, 1H), 7.58 (dd, *J* = 7.6, 2.0 Hz, 1H), 7.37-7.34 (m, 2H), 7.23 (dd, *J* = 8.4, 2.8 Hz, 1H), 3.89 (s, 3H), 3.29-3.25 (m, 4H), 2.92-2.83 (m, 1H), 1.95-1.91 (m, 2H), 1.65-1.61 (m, 2H). ^13^C NMR (100MHz, MeOD-*d4*) δ 164.4 (d, *J* = 238.3 Hz), 154.4, 148.6 (d, *J* = 13.9 Hz), 144.2 (d, *J* = 8.0 Hz), 140.7, 137.6, 136.8, 136.7, 135.9, 135.2 (d, *J* = 4.5 Hz), 125.9, 119.6, 116.7, 111.1, 110.5 (d, *J* = 36.8 Hz), 52.9, 33.9, 32.6, 32.3. HRMS (ESI) calcd. for C_21_H_20_FN_5_ [M + H]^+^ 362.1776, found: 362.1775. HPLC: t_R_ = 6.77 min, purity: 97.6%.

**2-(4-(1*H*-imidazol-5-yl)piperidin-1-yl)-3-(6-fluoropyridin-3-yl)benzonitrile** (**29**). Following similar procedures with compound **28**, 0.030g of the SEM protecting intermediate was obtained after purification on silica column. Then, the intermediate was dissolved in DCM (1 mL) and TFA (1 mL), and stirred overnight until the reaction was completed. The solvent was removed under reduced pressure, and the residue was dissolved in 7M NH_3_ in MeOH (1 mL), and stirred for an additional 3 hours. The mixture was concentrated again, purified using prep-HPLC and lyophilized to afford **29** as a white solid (11 mg, 31.7% for two steps). ^1^H NMR (400MHz, MeOD-*d4*) δ 8.83 (s, 1H), 8.29 (d, *J* = 2.4 Hz, 1H), 8.02 (td, *J* = 8.0, 2.4 Hz, 1H), 7.74 (dd, *J* = 8.0, 1.6 Hz, 1H), 7.57 (dd, *J* = 7.6, 1.6 Hz, 1H), 7.36-7.32 (m, 2H), 7.21 (dd, *J* = 8.4, 2.4 Hz, 1H), 3.29-3.24 (m, 2H), 3.16-3.11 (m, 2H), 2.88-2.79 (m, 1H), 1.98-1.93 (m, 2H), 1.69-1.65 (m, 2H). LC-MS (ESI) calcd. for C_20_H_19_FN_5_ [M + H]^+^ 348.2, found: 348.2. HPLC: t_R_ = 6.30 min, purity: 99.4%.

**Benzyl 4-(5-mercapto-4-methyl-4*H*-1,2,4-triazol-3-yl)piperidine-1-carboxylate** (**32**). To a solution of 1-[(benzyloxy) carbonyl] piperidine-4-carboxylic acid (**30**) (10.0 g, 38.0 mmol, 1.0 eq) in acetonitrile (80 mL), was added *N*, *N*-carbonyldiimidazole (6.16 g, 38.0 mmol, 1.0 eq) and the mixture was stirred at 50℃ for 2 hours. Then, *N*-methyl-thiosemicarbazide (**31**) (4.40 g, 41.8 mmol, 1.1 eq) was added, and the reaction mixture was stirred at 50℃ for additional 18 hours. The solvent was then removed under reduced pressure, and the residue was dissolved in dichloromethane and washed with a saturated NH_4_Cl solution. The organic phase was collected, and the resulting solution was concentrated under reduced pressure. The residue was purified via silica column, eluting with petroleum ether/ethyl acetate (1:1), which afford 9.80 g of the title compound (yield, 78%). ^1^H NMR (400MHz, MeOD-*d*_4_) δ 7.39-7.32 (m, 5H), 5.15 (s, 2H), 4.24-4.18 (m, 2H), 3.56 (s, 3H), 3.10-3.01 (m, 3H), 2.01-1.97 (m, 2H), 1.74-1.63 (m, 2H).

**Benzyl 4-(4-methyl-4*H*-1,2,4-triazol-3-yl)piperidine-1-carboxylate** (**33**). A solution of intermediate **32** (9.8 g, 29.7 mmol, 1.0 eq) in dichloromethane (20 mL) was cooled to 0℃, and hydrogen peroxide (30% water solution, 2.0 mL, 65.3 mmol, 2.2 eq) was added, followed by acetic acid (10 mL). The reaction mixture was allowed to warm up to room temperature and stirred for 18 hours. The reaction was then brought to pH 10 with 15% NaOH solution, and 30 mL of dichloromethane was added. The organic phase was collected, and the solvent was evaporated under reduced pressure. The resulting compound was purified by silica column chromatography using a SepaBean machine T200 to yield 8.1 g of the title compound (yield, 91%). ^1^H NMR (400MHz, CDCl_3_) δ 8.04 (s, 1H), 7.37-7.29 (m, 5H), 5.14 (s, 2H), 4.28 (d, *J* = 13.2 Hz, 1H), 3.64 (s, 3H), 3.02 (brs, 2H), 2.92-2.84 (m, 1H), 1.93 (brs, 4H).

**4-(4-Methyl-4*H*-[1,2,4]triazol-3-yl)-piperidine** (**34**). The intermediate **33** (8.1 g, 26.9 mmol, 1.0 eq) was dissolved in 6N HCl solution (25 mL), and the mixture was heated at 100 ℃ for 6 hours. After cooling the reaction mixture to room temperature, the aqueous phase was washed with 20 mL of dichloromethane and concentrated under reduced pressure, which afford 6.65 g of the title compound as hydrochloride salt. ^1^H NMR (400MHz, MeOD-*d_4_*) δ 9.65 (s, 1H), 4.01 (s, 3H), 3.60-3.54 (m, 3H), 3.32-3.26 (m, 2H), 2.37-2.33 (m, 2H), 2.20-2.08 (m, 2H).

**3-Bromo-2-(4-(4-methyl-4*H*-1,2,4-triazol-3-yl)piperidin-1-yl)benzonitrile** (**36**). The intermediate **34** (0.15 g, 1.21 mmol, 1.0 eq) was dissolved in *N*,*N*-dimethylformamide (4 mL) and heated at 100℃ for 18 hours under vigorous stirring, after adding potassium carbonate (0.25 g, 1.82 mmol, 1.5 eq) and 3-bromo-2-fluorobenzonitrile (**35**) (0.29 g, 1.45 mmol, 1.2 eq). After cooling the reaction mixture to room temperature, inorganic salts were filtered, and the resulting solution was concentrated under reduced pressure. The residue was suspended in dichloromethane and washed with water. The organic phase was collected and concentrated under reduced pressure. The residue was purified by silica column chromatography using SepaBean machine T200, which afford 0.15 g of the title compound (yield, 36%). ^1^H NMR (400MHz, MeOD-*d_4_*) δ 8.40 (s, 1H), 7.91 (d, *J* = 7.6 Hz, 1H), 7.66 (dd, *J* = 7.6, 2.4 Hz, 1H), 7.16 (t, *J* = 7.6 Hz, 1H), 3.79 (s, 3H), 3.63-3.56 (m, 2H), 3.43-3.32 (m, 2H), 3.17-3.08 (m, 1H), 2.26-2.15 (m, 2H), 2.10-2.05 (m, 2H).

***Tert*-butyl 4-(4-isopropyl-4*H*-1,2,4-triazol-3-yl)piperidine-1-carboxylate** (**38b**). To a solution of *tert*-butyl 4-(hydrazinecarbonyl)piperidine-1-carboxylate (**37**) (1.0 g, 4.1 mmol, 1.0 eq) in acetonitrile (10 mL), *N*,*N*-dimethylformamide dimethyl acetal (882 mg, 7.4 mmol, 1.8 eq) was added, and the mixture was stirred at 90 ℃ for 1 hours. Then, isopropylamine (786 mg, 14.8 mmol, 2.0 eq) was added, and the reaction was stirred at 90 ℃ for 18 hours. The solvent was removed under reduced pressure, and the residue was dissolved in dichloromethane and washed with a saturated NH_4_Cl solution. The organic phase was collected and concentrated under reduced pressure. The residue was purified by silica column chromatography, eluting with MeOH/DCM (0% - 5%), affording 723 mg of the title compound (yield, 60%). ^1^H NMR (400MHz, CDCl_3_) δ 8.23 (s, 1H), 4.35-4.28 (m, 1H), 2.83-2.75 (m, 5H), 1.92-1.78 (m, 4H), 1.48 (d, *J* = 6.8 Hz, 6H), 1.43 (s, 9H).

**4-(4-Isopropyl-4*H*-1,2,4-triazol-3-yl)piperidine** (**39b**). Tert-butyl 4-(4-isopropyl-4*H*-1,2,4-triazol-3-yl)piperidine-1-carboxylate (**38b**) (723 mg, 1.0eq) was dissolved in MeOH (4 mL), and 4M HCl in dioxane was added. The reaction mixture was stirred at room temperature overnight. The mixture was then concentrated under reduced pressure, affording 800 mg of the title compound as its hydrochloride salt.

**3-Bromo-2-(4-(4-isopropyl-4H-1,2,4-triazol-3-yl)piperidin-1-yl)benzonitrile** (**40b**). 4-(4-Isopropyl-4*H*-1,2,4-triazol-3-yl)piperidine (**39b**) (0.10 g, 0.5 mmol, 1.0 eq) was dissolved in *N,N*-dimethylformamide (4 mL), followed by addition of potassium carbonate (138 mg, 1.0 mmol, 2.0 eq) and 3-bromo-2-fluorobenzonitrile (**35**) (0.15 g, 0.75 mmol, 1.5 eq). The reaction mixture was heated at 100 ℃ for 18 hours with vigorous stirring. After cooling to room temperature, the inorganic salts were filtered, and the resulting solution was concentrated under reduced pressure. The residue was suspended in DCM and washed with water. The organic phase was collected and concentrated under reduced pressure. The residue was purified by silica column using SepaBean machine T200, and 105 mg of the title compound was obtained (yield, 60%).^1^H NMR (400MHz, CDCl_3_) δ 8.20 (s, 1H), 7.80 (d, *J* = 8.0 Hz, 1H), 7.52 (dd, *J* = 8.0, 1.6 Hz, 1H), 7.01 (t, *J* = 8.0 Hz, 1H), 4.43-4.36 (m, 1H), 3.54-3.41 (m, 4H), 2.91-2.84 (m, 1H), 2.42-2.31 (m, 2H), 2.01-1.97 (m, 2H), 1.51 (d, *J* = 6.8 Hz, 1H).

**3-Bromo-2-(4-(1-methyl-1H-imidazol-5-yl)piperidin-1-yl)benzonitrile** (**42a**): 4-(1-methyl-1H-imidazol-5-yl)piperidine (**41a**) (0.15g, 1.21 mmol, 1.0 eq) was dissolved in *N,N*-dimethylformamide (4 mL), followed by addition of potassium carbonate (0.25 g, 1.82 mmol, 1.5 eq) and 3-bromo-2-fluorobenzonitrile (**35**) (0.29 g, 1.45 mmol, 1.2 eq). The reaction mixture was heated at 100 ℃ for 18 hours with vigorous stirring. After cooling to room temperature, the inorganic salts were filtered, and the resulting solution was concentrated under reduced pressure. The residue was suspended in DCM and washed with water. The organic phase was collected and concentrated under reduced pressure. The residue was purified by silica column using SepaBean machine T200, and 0.15 g of the title compound was obtained (yield, 29%).^1^H NMR (400MHz, MeOD-*d_4_*) δ 7.90 (d, *J* = 7.6 Hz, 1H), 7.66 (d, *J* = 8.0 Hz, 1H), 7.58 (s, 3H), 7.15 (t, *J* = 7.2 Hz, 1H), 6.82 (s, 3H), 3.72 (s, 3H), 3.61-3.54 (m, 2H), 3.43-3.37 (m, 3H), 2.08-2.04 (m, 2H), 1.99-1.91 (m, 2H). LRMS (ESI) [M + H]^+^, found: 347.0.

Cell culture.

HEK293T, B16F10, HCT116, SKOV3, Huh7, T24 and MCF-6 cells were cultured in DMEM supplemented with 10% fetal bovine serum (FBS, Hyclone), 100 U/ mL penicillin, and 100 μg/ mL streptomycin (Invitrogen) at 37 °C in 5% CO_2_, SU-DHL-8, H929, Raji, H1299, Jurkat cells were cultured in RPMI 1640 supplemented with 10% FBS, 100 U/ mL penicillin, and 100 μg/ mL streptomycin at 37 °C in 5% CO_2_.

PGPEP I protein purification.

The plasmid of human PGPEP I (pyroglutamyl aminopeptidase I) was a kind gift from Professor Haiqiang Wu of Shenzhen University. After confirmation of the plasmid, the DNA fragment was inserted into the pET28a expression vector. Subsequently, the vector containing PGPEP I was transformed into E. Coli BL21 (DE3) cells (Tsingke Biotechnology Co., Ltd.), and then the bacteria were grown in LB media with 100 μg/mL kanamycin at 37°C until an OD_600_ of ≈ 0.8 was reached. The cultures were induced with 1 mM isopropyl β-D-thiogalactopyranoside (IPTG) at 20°C for 48 hours. The cells were collected and resuspended in balance buffer (50 mM Tris-HCl, 150 mM NaCl, pH= 8). After clarifying by centrifugation (10000 rpm for 5 min at 4 °C), the cells were resuspended in balance buffer added with 1 mM PMSF, DNase and Mg^2+^ followed by high-pressure homogenization, centrifuging the lysate at 12000 rpm for 30 min at 4 °C. The purification was initiated by Ni-NTA beads. The bound PGPEP I was eluted with balance buffer containing 300 mM imidazole. The PGPEP I fraction was pooled and further purified using a SuperdexTM 200 Increase 10/300 GL column (Cytiva) with balance buffer. The PGPEP I containing fractions were collected and concentrated by ultrafiltration tube, purified protein was stable for months in 20% glycerol at -80 °C.

QPCTL protein purification.

The DNA with sequence coding for the human glutaminyl-peptide cyclotransferase-like protein (nucleotide entry: NP_060129) was synthesized and then inserted into the pGEX-4T-2 vector. The vector was transformed into Escherichia coli BL21 (DE3) competent cells (Tiangen Biotech, Cat. # CB105). The bacteria were grown in LB media containing ampicillin (100 μg/ mL) at 37 °C until the cell density reached an OD_600_ of 0.8- 0.9. The cultures were induced with 1 mM IPTG for 8- 10 h at 20 °C. The bacteria were collected and resuspended in 50 mL PBS, and 1% Triton X-100 (v/v), 1% β-mercaptoethanol (v/v), PMSF (final concentration 1 mM) were added into the mixed solution. The bacterial cells were then harvested by centrifugation (4,700 g for 30 min at 4 °C) followed by ultrasonic breaking. The resulted solution was clarified by centrifugation at 13,800 g for 30 min. GST-beads was suspended to the supernatant and was gently shook for 1 hour to bind the protein. The resulted protein solution was centrifuged at 1,500 g for 5 min and the supernatant was discarded. At least 10-fold the volume of PBS was added to the pellet to sufficiently suspend the beads in the solution. Then the solution was clarified by centrifugation at 1,500 g for 5 min and the supernatant was discarded. The above steps were repeated twice. 1 mL GST elution buffer was added to the pellet and the solution was shaken gently for 30 min following by centrifugation at 1,500 g for 5 min. The supernatant was collected and was eluted with GST elution buffer at least twice. The supernatant was loaded onto SDS-PAGE electrophoresis to detect protein purity. The protein was stored in 20% glycerol at -80 °C.

Enzyme activity assay.

Enzyme activity was measured by conversion of glutamine-4-amino-7-methylcounarine (H-Gln-AMC) to pyroglutamyl-AMC, which is subsequently a specific substrate for PGPEP I, releasing free AMC which can be fluorescently detected. For typical inhibition assay, 12.5 μL QPCTL protein (7 ng/μL) or QPCT protein (7 ng/μL) (MedChemExpress, Cat. #HY-P76560) and 2.5 μL of tested compounds with different concentrations were pipetted into a 384-well plate on ice. The reaction system was reacted in a shaker at 37 °C, 100 rpm for 10 minutes. Next, 10 μL fluorescent substrate H-Gln-AMC (500 μM) was added and incubated in 100 rpm for 20 min at 37 °C. Finally, 25 μL PGPEP I (5.74 ng/μL) was added to the system and incubated at 37 °C in 100 rpm for 30 min. Once done, the plate was moved over to TECAN Infinite 200PRO plate-reader to monitor and record the maximum fluorescence at 380nm excitation/ 460nm emission. The fluorescent signal value obtained was analyzed by Graphpad Prism 8.0 to obtain the inhibitory rate and IC_50_ of the compound.

Isolation of bone marrow-derived macrophages.

Eight-week-old C57BL/6 wild-type mice were obtained from Shanghai Slaccas Laboratory Animal Co., Ltd.. To obtain bone marrow derived macrophage (BMDM), femora and tibiae were flushed with ice-cold PBS, and erythrocytes were lysed with RBC lysis buffer. Then bone marrow cells were cultured in DMEM medium with 10 % FBS and 100 U/ mL penicillin, and 100 μg/ mL streptomycin in the presence of 50 ng/ mL mouse M-CSF (Macrophage-Colony Stimulating Factor) (PeproTech), 0.1 mM Sodium pyruvate (Gibco, Cat. # 11360-70) and 10 mM HEPES (Gibco, Cat. # 15630-080) for 7 days.

Flow cytometry assay.

Binding to cell surface CD47 was assessed by staining cells with fluorochrome-labeled antibodies clone CC2C6 (BioLegend, Cat. # 323102) and B6H12 (Abcam, Cat. # ab134485) to human CD47 at a dilution of 1:100 in PBS containing 1% FBS (FACS buffer) for 30 min on ice, while protected from light. SIRPα binding to CD47 was assessed by incubating cells with PE labeled recombinant human SIRPα/ CD172α Protein, Fc tag (ACRO Biosystems, Cat. # SIA-HP252) or PE labeled mouse SIRPα Protein, His tag (ACRO Biosystems, Cat. # SIA-MP2H6) for 1 hour on ice, while protected from light.

Phagocytosis assay were conducted as previously described. Briefly, macrophages were plated 1 x 10^^5^ cells per well in a 48-well plate in complete DMEM medium, supplemented with M-CSF overnight before the experiment. Cancer cells were stained with 5 μM CFSE at 37 °C for 10 min. Each phagocytotic reaction reported in this work was performed by co-culture of target cells and macrophages for 4 hours at 37 °C. Macrophages were identified with APC-labeled anti-F4/80 (Biolegend, Cat. # 123116), and flow cytometry (BD FACSCanto II, San Jose, CA) was performed. Phagocytosis was calculated as the percentage of CFSE^+^F4/80^+^ cells (Q2) among CFSE^+^ cells (Q2+Q3): phagocytosis (%) = [Q2 / (Q2+ Q3)] x100 %.

Efferocytosis

Apoptotic corpse generation. Jurkat cells were cultured in a T25 flask and apoptotic corpses were inducted by exposure to 5 μM camptothecin (Tsbiochem, CAT. # t2764) for 4 hours. Apoptosis induction was confirmed by apoptoic kit (Beyotime Biotechnology, Cat. # C1062M) through flow cytometry.

Senescence induction. B16F10 Cells were plated (96-well plate: 2000 cells/well) and rested over night before exposure to 10 μM cisplatin (APExBIO, Cat. # A8321). Cisplatin exposed cells were subsequently cultured for 4 or 7 days (without media change) and senescent cells were evaluated by β-Galactosidase staining using a SA-β-Gal staining Kit (Solarbio, Cat. # G1580-100T) according to the manufacturer’s instructions. Senescent cells were identified as blue stained cells under light microscopy.

Efferocytosis after co-culture. Senescent cells were plated in 96-well plates (2000 cells/well) and inducted as described before. QPCTL inhibitors were added every other day to the cells. For co-culture conditions BMDMs were added to the senescent cells. Senescent cells and macrophages were co-cultured for 16 hours. Apoptotic Jurkat corpses were labeled with CFSE as described before and were then incubated with the macrophages for 60 min at a 1:2 ratio (macrophages: Jurkat). Following removal of unbound corpses by washing three times with PBS, macrophages were marked with APC-labeled anti-F4/80, and the co-culture was analyzed by flow cytometry. Efferocytosis was calculated as the percentage of CFSE^+^F4/80^+^ cells among F4/80^+^ cells.

Cell viability.

Raji cells (5000 cells/well), B16F10 cells (3000 cells/well) and BMDM (10000 cells/well) were plated in 96-well and cell viability was examined at the time points shown in the figures, with the help of Cell Counting Kit 8 (CCK-8) (Share-bio, Cat. # B-CCK8) according to the manufacturer’s recommendations.

Molecular docking studies.

All the procedure was performed using Maestro software (Schrodinger LLC). The crystal structure of human QPCTL was taken from protein database bank (PDB ID: 3PB7) and the crystal structure of human QPCT was taken from protein database bank (PDB ID: 6GBX). Then, the protein was processed using the ‘Protein Preparation Wizard’ workflow in Maestro to adding bond orders and hydrogens. All het atm residues and crystal water molecules beyond 5 Å from het group were removed. The co-crystallized molecule was removed from the system. QPCTL inhibitor was built by LigPrep module by OPLS-2005 force field. Glide module was used as docking program. The active site was defined using the zinc as the center. The docking box was placed on the centroid of the binding ligand in the optimized crystal structure as described above.

Tumor challenge.

C57BL/6 mice were obtained from Shanghai Slaccas Laboratory Animal Co., Ltd.. Male mice were implanted subcutaneously with 1 x 10^^6^ B16F10 cells resuspended in 100μL of DMEM on Day 0. Anti-PD-1 (Clone RMP1-14, BioXCell, Cat. # BE0146) or mouse IgG2a isotype control (Clone 2A3, BioXCell, Cat. # BE0089) was dosed at 80 μg per mouse intraperitoneally starting on day 12 after tumor implantation, followed by a once-a-week regimen. QPCTL inhibitor was intraperitoneally administered at 25 mg/kg once a day. Tumors were measured daily, and mice were sacrificed 24 days after tumor challenge. Tumor volumes were measured in two dimensions (length and width) and the volume was calculated using the formula, tumor size (mm^3^) = (length × width^2^) × 0.5.

Tumor challenge compared to reported QPCTL inhibitor SEN177. Briefly, male mice were implanted subcutaneously with 5 x 10^5 B16F10 cells resuspended in 100 μL of DMEM on Day 0. Anti-PD-1 antibody or mouse IgG2a isotype control was dosed at 80 μg per mouse intraperitoneally starting on day 6 after tumor implantation, followed by a once-a-week regimen. QPCTL inhibitors was intraperitoneally administered at 25 mg/kg once a day since day 6. Tumors were measured every three days, and mice were sacrificed on day 15. Mouse organs and blood were collected for blood routine, blood serum biochemistry examination and histological evaluation.

Blood routine and blood serum biochemistry examination of mice. Whole blood was collected in EDTA anticoagulant tubes for blood routine test and heparin sodium tubes for blood serum biochemistry examination. Tubes were gently inverted several times to thoroughly mix the blood and anticoagulant. The routine blood test was tested by Automatic Hematology Analyzer (TEK8500H4-0502, TECOM SCIENCE, China). Blood serum was obtained by centrifugation at 4℃ at 4000 rpm for 10 minutes. Serum creatinine (CR) was determined by CR kit (PUREBIO, Cat. # CR01), urea nitrogen (BUN) was determined by BUN kit (PUREBIO, Cat. # URE01), alanine aminotransferase (ALT) was determined by ALT kit (PUREBIO, Cat. # ALT01) and alanine aminotransferase was determined by AST kit (PUREBIO, Cat. # AST01) according to the manufacturer’s instructions.

Histological examination of C57B/L6 mice by hematoxylin and eosin (HE) staining. Mice liver, kidney, spleen and heart were immersed in 4% paraformaldehyde for 4 hours, and transferred to 70% ethanol. Individual organ biopsy materials were placed in processing cassettes, dehydrated through a serial alcohol gradient, and embedded in paraffin wax blocks. Before immunostaining, 5μm-thick lung tissue sections were dewaxed in xylene, rehydrated through decreasing concentrations of ethanol, and washed in PBS. Then they were stained with hematoxylin and eosin (H&E, Recordbio, Cat. # RCF040-Red). Sections were dehydrated through increasing concentrations of ethanol and xylene. All images were collected with a microscope (NIKON ECLIPSE CI-L).

Tumor challenge with T cell depletion. 6-8 week-old male C57BL/6 mice were subcutaneously injected with B16F10 cells (8.5 x 10^^5^/ 100 µL) into the right flank. The T cell depletion was started one day (Day -1) before tumor implantation (Day 0). 200 µg/ mouse of the anti-CD3 antibody (BioXcell, Cat. # BE0001-1-25MG) or isotype (BioXcell, Cat. # BE0091-25MG) were injected to the tumor cavities every other 4 days. When tumor volume reached approximately 50 mm^3^, mice were intraperitoneally injected first dose anti-PD-1 antibody and **QP5038**/ vehicle control. **QP5038** was daily dosed 25 mg/kg per mouse and anti-PD-1 antibody was dosed 80 µg/ mouse once a week. Animal weights and tumor volumes were measured twice weekly throughout the study. The experiments were stopped when the tumor diameter reached 2 cm. T cell depletion was confirmed by flow cytometry. At the end of experiment, the mice were sacrificed and tumor were collected. After lysing red blood cells with lysing buffer, tumor cells were resuspended in FACS buffer. A single-cell suspension was treated with PE-anti-mouse CD3 antibody (BioLegend, Cat. # 100205; 1:200) on ice in the dark for 30 min. Data were acquired on flow cytometry analyzer and analyzed using Flowjo software.

Statistical analysis.

GraphPad Prism 8.0 software was used for data analysis. All experiments were repeated at least three times. Data were shown as mean ± SD. Statistical significance was evaluated by Student’s t-test, one-way ANOVA or two-way ANOVA. P value was considered statistically significant.

References.

1 Jimenez-Sanchez, M. et al. siRNA screen identifies QPCT as a druggable target for Huntington's disease. *Nat. Chem. Biol.* **11**, 347-354, (2015).
